# AMPK and glucose deprivation exert an isoform-specific effect on the expression of Na^+^,K^+^-ATPase subunits in cultured myotubes

**DOI:** 10.1007/s10974-024-09673-9

**Published:** 2024-05-06

**Authors:** Anja Vidović, Klemen Dolinar, Alexander V. Chibalin, Sergej Pirkmajer

**Affiliations:** 1 Faculty of Medicine, Institute of Pathophysiology, University of Ljubljana, Ljubljana, Slovenia; 2 Department of Molecular Medicine and Surgery, Integrative Physiology, Karolinska Institutet, Stockholm, Sweden; 3 National Research Tomsk State University, Tomsk, Russia

**Keywords:** Na^+^,K^+^-ATPase, AMPK, Energy stress, Glucose, Myotubes, Skeletal muscle

## Abstract

In skeletal muscle, Na^+^,K^+^-ATPase (NKA), a heterodimeric (α/β) P-type ATPase, has an essential role in maintenance of Na^+^ and K^+^ homeostasis, excitability, and contractility. AMP-activated protein kinase (AMPK), an energy sensor, increases the membrane abundance and activity of NKA in L6 myotubes, but its potential role in regulation of NKA content in skeletal muscle, which determines maximum capacity for Na^+^ and K^+^ transport, has not been clearly delineated. We examined whether energy stress and/or AMPK affect expression of NKA subunits in rat L6 and primary human myotubes. Energy stress, induced by glucose deprivation, increased protein content of NKAα1 and NKAα2 in L6 myotubes, while decreasing the content of NKAα1 in human myotubes. Pharmacological AMPK activators (AICAR, A-769662, and diflunisal) modulated expression of NKA subunits, but their effects only partially mimicked those that occurred in response to glucose deprivation, indicating that AMPK does not mediate all effects of energy stress on NKA expression. Gene silencing of AMPKα1/α2 increased protein levels of NKAα1 in L6 myotubes and NKAα1 mRNA levels in human myotubes, while decreasing NKAα2 protein levels in L6 myotubes. Collectively, our results suggest a role for energy stress and AMPK in modulation of NKA expression in skeletal muscle. However, their modulatory effects were not conserved between L6 myotubes and primary human myotubes, which suggests that coupling between energy stress, AMPK, and regulation of NKA expression in vitro depends on skeletal muscle cell model.

**Supplementary Information:**

The online version contains supplementary material available at 10.1007/s10974-024-09673-9.

## Introduction

Na^+^,K^+^-ATPase (NKA), the Na^+^-K^+^ pump, counteracts the uptake of Na^+^ and the loss of K^+^ ions, thus maintaining excitability and contractility of skeletal muscle (reviewed in (Clausen 2003)). The basic functional unit of NKA is a heterodimer of a catalytic α-subunit (NKAα), a 100–112 kDa type IIC P-ATPase, and a non-catalytic, 35–60 kDa glycoprotein, β-subunit (NKAβ) (Ackermann and Geering 1990; McDonough et al. 1990; Geering 2008). Of the four NKAα isoforms (NKAα1–4) (Blanco and Mercer 1998), skeletal muscle expresses almost exclusively NKAα2 (~ 60–90%) and NKAα1 (Orlowski and Lingrel 1988; Sweadner et al. 1992; Hansen 2001; He et al. 2001; Kristensen and Juel 2010), which play specific roles in maintaining ion homeostasis in contracting (NKAα2) or resting (NKAα1) skeletal muscle (Radzyukevich et al. 2004, 2013). All three β-subunits (NKAβ1–3), which are essential for NKA assembly, maturation, and function (Ackermann and Geering 1990; McDonough et al. 1990; Geering 2008), are expressed in skeletal muscle (Boon et al. 2012; Perry et al. 2013; Christiansen et al. 2018), with NKAβ1 and β2 being best characterized (reviewed in: (Christiansen 2019; Wyckelsma et al. 2019)). The kinetic properties of NKA are modulated by FXYD proteins (FXYD1-7), which are often considered as a third, ancillary γ-subunit (Sweadner and Rael 2000; Geering 2006, 2008; Morth et al. 2007). In skeletal muscle, FXYD1 (phospholemman) (Palmer et al. 1991), which suppresses NKA activity by decreasing NKA affinity for Na^+^ (Crambert et al. 2002), is functionally most important (reviewed in: (Pirkmajer and Chibalin 2016; Christiansen 2019)), but data in humans (Boon et al. 2012; Hostrup et al. 2023) and cultured myotubes (Jan et al. 2021) suggest that FXYD5 (dysadherin), which increases NKA *V*_*max*_ (Lubarski et al. 2005, 2007), also plays a role.

Contractions activate NKA, whereas its inhibition with ouabain accelerates the loss of force of the contracting muscle (Nielsen and Clausen 1996, 1997), emphasising the importance of a tight coupling between contractile and NKA activities in skeletal muscle. The rate of Na^+^-K^+^ transport at any given time depends on the number and activity of NKA units in the plasmalemma, whereas the maximal Na^+^-K^+^ transport capacity is determined by the total content of NKA units in the muscle (Nielsen and Clausen 1997). This includes NKA located in intracellular vesicles (Benziane and Chibalin 2008), which can serve as an acute source of additional NKA units for the plasmalemma. By stimulating the translocation of vesicular NKA into the plasmalemma, contractions increase the number of NKA units in the membrane, presumably contributing to the activation of Na^+^-K^+^ transport in contracting muscles (Benziane and Chibalin 2008; Kristensen et al. 2008) (reviewed in: (Pirkmajer and Chibalin 2016)). Muscle contractions also promote expression of NKA, with regular physical activity leading to an increase in the total NKA content in skeletal muscle (reviewed in: (Clausen 2003)). The content of NKA does not directly correlate with NKA activity, but it determines the maximal capacity to perform Na^+^-K^+^ transport (Nielsen and Clausen 1997), thus affecting the ability of skeletal muscle to maintain ion homeostasis during contractions.

Contractions activate AMP-activated protein kinase (AMPK) (Winder and Hardie 1996) (reviewed in: (Kjobsted et al. 2018; McConell 2020)), an energy gauge, which maintains energy balance by inhibiting ATP-consuming processes and stimulating ATP-producing processes (Hardie et al. 2012a, b; Hardie 2018). Activation of AMPK decreased membrane abundance and NKA activity in lung alveolar cells (Woollhead et al. 2005; Woollhead and Baines 2006; Vadasz et al. 2008; Gusarova et al. 2009), as would be expected in an ATP-consuming process, but the regulation of NKA by AMPK appears to be different in skeletal muscle (reviewed in: (Pirkmajer et al. 2021)). In rats, acute treatment with the AMPK activator AICAR did not decrease NKA membrane abundance and/or activity in skeletal muscle (Lemieux et al. 2003; Zheng et al. 2008). Moreover, AICAR increased NKA activity in rat L6 myotubes by decreasing the internalization of NKA (Benziane et al. 2012), implicating a role for AMPK in acute regulation of NKA. However, AMPK is particularly important for adaptations that occur in skeletal muscle following contraction (Kjobsted et al. 2019; McConell 2020), which raises the question whether AMPK might modulate expression and content of NKA in skeletal muscle.

In humans, exercise with blood flow restriction, which induces sufficient energy stress to activate AMPK (Christiansen et al. 2018), upregulated NKAα1, NKAβ1, and/or FXYD1 (Christiansen et al. 2018, 2019). On the other hand, chronic treatment with AICAR did not alter the membrane abundance of NKAα and NKAβ subunits in mice, but it decreased the amount of total FXYD1 (Ingwersen et al. 2011). Further, in the AMPK kinase-dead mice, FXYD1 was upregulated, without any change in the abundance of NKAα and NKAβ (Ingwersen et al. 2011). Taken together, these studies seem to suggest that while energy stress affects expression and content of various NKA subunits in skeletal muscle, AMPK regulates only expression of FXYD1. However, under in vivo conditions interaction between multiple local and systemic responses could have contributed to the observed results, meaning that the role of energy stress and AMPK in regulation of NKA expression in skeletal muscle requires further characterization.

In the current study we used cultured myotubes to determine (1) whether energy stress, induced by glucose deprivation, modulates expression of NKA subunits, (2) whether AMPK affects the expression of NKA subunits, and (3) whether energy stress, induced by glucose deprivation, modulates expression of NKA subunits in an AMPK-dependent manner.

## Materials and methods

### Materials and reagents

Cell culture flasks and plates were from Sarsted or TPP. MEMα without nucleosides (#22561), Advanced MEM (#12492), Dulbecco’s Modified Eagle Medium (DMEM) – without glucose (#11966), DMEM-Glutamax – 1 g/L glucose (#21855), DMEM-Glutamax – 4.5 g/L glucose (#31966), foetal bovine serum (FBS), Pen Strep (5,000 U/mL penicillin and 5,000 µg/mL streptomycin), Fungizone (250 µg/mL of amphotericin B), gentamicin (10 mg/mL), Pierce BCA Protein Assay Kit, Pierce 660 nm Protein Assay Reagent, Pierce Enhanced Chemiluminescence (ECL) Western Blotting Substrate, High-Capacity cDNA Reverse Transcription Kit and TaqMan Universal Master Mix were from ThermoFisher Scientific. 96-well reaction plates and adhesive films were also from ThermoFisher Scientific or Bio-Rad. The E.Z.N.A. HP Total RNA Kit was from Omega Bio-Tek. MACS CD56 MicroBeads were from Miltenyi Biotec. The 4–12% Criterion XT Bis-Tris polyacrylamide gels, XT MES electrophoresis buffer, goat anti-rabbit IgG horseradish peroxidase conjugate (#170–6515), and goat anti-mouse IgG horseradish peroxidase conjugate (#170–6516) were from Bio-Rad (Hercules, CA, U.S.). The Amersham ECL Full-Range Rainbow Molecular Weight Markers were from Cytiva. 5-aminoimidazole-4-carboxamide-1-β-D-ribofuranoside (AICAR) (#10010241) was from Cayman Chemical, A-769662 (#1466) from Axon Medchem, and D(+)-glucose (#16325) from Riedel-de Haën. Polyvinylidene fluoride (PVDF) membrane, D-mannitol (#M4125), and all other reagents were from Merck/Sigma-Aldrich (Darmstadt, Germany) unless otherwise indicated. The primary antibodies and gene expression assays are listed in Tables 1 and 2, respectively.

**Table 1:** Gene expression assays

Species	Target	Gene	Cat. No.
*Homo sapiens*	18S rRNA	*18S *	Hs99999901_s1
	AMPKα1	*PRKAA1*	Hs01562315_m1
	AMPKα2	*PRKAA2*	Hs00178903_m1
	NKAα1	*ATP1A1*	Hs00167556_m1
	NKAα2	*ATP1A2*	Hs00265131_m1, Hs01560077_m1
*Rattus norvegicus*	AMPKα1	*Prkaa1*	Rn00665045_m1
	AMPKα2	*Prkaa2*	Rn00576935_m1
	FXYD1 (phospholemman)	*Fxyd1*	Rn00581299_m1
	NKAα1	*Atp1a1*	Rn01533986_m1
	NKAα2	*Atp1a2*	Rn00560789_m1
	NKAβ1	*Atp1b1*	Rn00565405_m1

**Table 2:** Antibodies used for immunoblotting

	Primary antibody			Secondary antibody	
Target protein	Species	Dilution	Cat. No.	Species	Dilution
ACC	rabbit	1:1,000	#3676 (CST)	GAR	1:15,000
AMPKα	rabbit	1:1,000	#2532 (CST)	GAR	1:10,000
AMPKα1	rabbit	1:1,000	#5832S (CST)	GAR	1:15,000
AMPKα2	rabbit	1:1,000	#2757S (CST)	GAR	1:10,000
AMPKα2	rabbit	1:1,000	#PA5-21494 (TF)	GAR	1:10,000
FXYD1	rabbit	1:500	#13721-1-AP (TF)	GAR	1:10,000
NKAα1	mouse	1:2,000	#05-369 (Merck)	GAM	1:30,000
NKAα2	rabbit	1:4,000	#AB9094-1 (Merck)	GAR	1:10,000
NKAβ1	mouse	1:2,000	#MA3-930 (TF)	GAM	1:30,000
NKAβ1	mouse	1:3,000	#MA1-16732 (TF)	GAM	1:20,000
Phospho-ACC (Ser79)	rabbit	1:1,000	#3661 (CST)	GAR	1:15,000
Phospho-AMPKα (Thr172)	rabbit	1:1,000	#2535 (CST)	GAR	1:15,000

*Abbreviations* CST – Cell Signaling Technology; TF - Thermo Fisher Scientific; GAM – goat anti-mouse IgG-HRP conjugate #1706516 from Bio-Rad; GAR – goat anti-rabbit IgG-HRP conjugate #1706515 from Bio-Rad

### L6 skeletal muscle cells

The rat L6 skeletal muscle cell line was obtained from ATCC. L6 cells were cultured as described (Dolinar et al. 2018). During myoblast proliferation and differentiation into myotubes medium always contained 1 g/L glucose. In brief, L6 myoblasts were cultured in MEMα (with nucleosides and 1 g/L glucose) supplemented with 10% (v/v) FBS, 1% (v/v) Pen Strep (50 U/mL penicillin and 50 µg/mL streptomycin) and 0.3% (v/v) Fungizone (0.75 µg/mL amphotericin B) in humidified atmosphere with 5% (v/v) CO_2_. To obtain differentiated myotubes from myoblasts, the growth medium was changed to a differentiation medium (MEMα (with nucleosides and 1 g/L glucose) supplemented with 2% (v/v) FBS, 1% (v/v) Pen Strep (50 U/mL penicillin and 50 µg/mL streptomycin) and 0.3% (v/v) Fungizone (0.75 µg/mL amphotericin B)) for 7–10 days. To determine effect of glucose on NKA subunit expression, myotubes were exposed to serum-free DMEM with different glucose concentrations for 24 h as indicated in the Results. In this case, the experiments were performed in DMEM because glucose-free MEMα is not commercially available. Experiments with AMPK activators were performed in serum-free MEMα (1 g/L glucose) without nucleosides, FBS and antibiotics. In this case, L6 myotubes were treated with AICAR, A-769662, or diflunisal for 24 h in MEMα (1 g/L glucose) without nucleosides, FBS, and antibiotics.

### Primary human skeletal muscle cell cultures

The preparation and use of primary human skeletal muscle cells (HSMC) was approved by the Republic of Slovenia National Medical Ethics Committee (ethical approval no. 71/05/12 and 0120–698/2017/4). HSMCs were prepared as described (Pirkmajer et al. 2020; Jan et al. 2021). During myoblast proliferation and differentiation into myotubes medium always contained 1 g/L glucose. In brief, primary skeletal muscle cultures were prepared from the *semitendinosus* muscle samples obtained as surgical waste during reconstructive surgery of the anterior cruciate ligament of the knee. All donors signed an informed consent. The muscle tissue was cleaned of connective and adipose tissue, cut into small pieces, and trypsinised. The released cells were grown in Advanced MEM (1 g/L glucose) supplemented with 10% (v/v) FBS, 0.3% (v/v) Fungizone (0.75 µg/mL amphotericin B) and 0.15% (v/v) gentamicin (15 µg/mL) in a humidified atmosphere with 5% (v/v) CO_2_. Skeletal muscle cells were purified before reaching confluence with MACS CD56 MicroBeads as described (Jan et al. 2021). Experiments were performed on uncoated or Matrigel (#356231, Corning) coated plates. To initiate differentiation into myotubes, myoblasts were placed in the differentiation medium (Advanced MEM (1 g/L glucose) supplemented with 2% (v/v) FBS, 0.3% (v/v) Fungizone (0.75 µg/mL of amphotericin B) and 0.15% (v/v) gentamicin (15 µg/mL)). During the last 48 h before the experiment, myotubes were cultured in DMEM (1 g/L glucose) supplemented with 2% (v/v) FBS. To determine effect of glucose on NKA subunit expression, myotubes were exposed to serum-free DMEM with different glucose concentrations for 24 h as indicated in the Results.

### Gene silencing of AMPKα1 and AMPKα2

L6 cells or HSMC were seeded on 12-well cell culture plates, which were coated with Matrigel for HSMC. Gene silencing was performed on day 3 and 5 of differentiation for L6 cells and on day 2 of differentiation for HSMC. L6 cells and HSMC were switched to differentiation medium without antibiotics and antimycotics 1 h (L6 cells) and 4 h (HSMC) before transfection. L6 cells were transfected with 26 nM of siRNAs against rat AMPK α1 and α2 mRNA (*Prkaa1* and *Prkaa2*, #L-091373-02-0005 and #L-100623-02-0005, ON-TARGETplus–SMARTpool, Dharmacon Horizon Discovery), while HSMC were transfected with 21 nM of siRNAs against human AMPK α1 and α2 mRNA (*PRKAA1* and *PRKAA2*, #L-005027-00-0005 and #L-005361-00-0005, ON-TARGETplus–SMARTpool, Dharmacon Horizon Discovery). To test the off-target effects of siRNA, cells were treated with 52 nM (L6 cells) or 42 nM (HSMC) of non-targeting siRNA (ON-TARGET plus Non-targeting Pool, #D-001810-10-20, Dharmacon Horizon Discovery). Transfections were performed using X-tremeGENE 360 Transfection Reagent (#08724121001, Roche) according to the manufacturer’s protocol. The siRNA complexes were prepared in Opti-MEM (Thermo Fisher Scientific) and added to the cells in differentiation medium without antibiotics and antimycotics. After 24 h, the medium was removed and replaced with fresh differentiation medium which contained antibiotics and antimycotics. The experiments were performed on day 8 of differentiation for L6 cells and on day 7 for HSMC.

### Quantitative real-time PCR (qPCR)

Myotubes were washed with ice-cold phosphate-buffered saline (PBS: 137 mM NaCl, 2.7 mM KCl, 10 mM Na_2_HPO_4_, 1.8 mM KH_2_PO_4_, pH 7.4) and total RNA was isolated using the E.Z.N.A. HP Total RNA Kit. Reverse transcription was performed using the High-Capacity cDNA Reverse Transcription Kit. QPCR was performed with the 7500 Real-Time PCR System and QuantStudio3 (Thermo Fisher Scientific) using TaqMan gene expression assays from Thermo Fisher Scientific (Table 1). Results are expressed as gene expression ratio: *(1 + E*_*reference*_*)*^*Ct, reference*^*/(1 + E*_*target*_*)*^*Ct, target*^, where E is the PCR efficiency and Ct is the threshold cycle. PCR efficiency was estimated using LinRegPCR software (Ruijter et al. 2009; Tuomi et al. 2010). 18S rRNA was used as a reference gene. To evaluate the stability of the reference gene, RefFinder, an online-based analysis tool (Xie et al. 2012), was used.

### Immunoblotting

Myotubes were washed with ice-cold PBS and lysed in Laemmli buffer (62.5 mM Tris-HCl (pH 6.8), 10% (w/v) glycerol, 2% (w/v) sodium dodecyl sulphate (SDS), 5% (v/v) 2-mercaptoethanol, 0.002% (w/v) bromophenol blue), as described (Dolinar et al. 2018; Pirkmajer et al. 2020; Jan et al. 2021). Protein concentrations were measured using the Pierce 660 nm protein assay. An equal mass of total protein was loaded onto a 4–12% polyacrylamide gel and resolved by SDS-PAGE in XT MES buffer at 200 V. Proteins were transferred to a PVDF membrane at 100 V in transfer buffer (31 mM Tris, 0.24 M glycine, 10% (v/v) methanol, and 0.01% (w/v) SDS) using the Criterion system (Bio-Rad). After transfer, the membranes were stained with Ponceau S (0.1% (w/v) in 5% (v/v) acetic acid) to assess loading and transfer efficiency. The membranes were then blocked with 7.5% (w/v) dry milk in Tris-buffered saline with Tween (TBST: 20 mM Tris, 150 mM NaCl, 0.02% (v/v) Tween 20, pH 7.5) for 1 h at room temperature. After blocking, the membranes were incubated with primary antibodies (Table 2) at 4 °C overnight. The primary antibodies were diluted in the primary antibody buffer (20 mM Tris, 150 mM NaCl, 0.1% (w/v) BSA, pH 7.5 and 0.1% (w/v) NaN_3_). The membranes were then washed with TBST and incubated with the secondary antibodies conjugated with horseradish peroxidase in TBST with 5% (w/v) dry milk for 1 h at room temperature. The immunolabelled proteins (bands) were visualised using Fusion FX from Vilber and analysed using Quantity One 1-D Analysis Software 4.6.8. (Bio-Rad).

### Assessment of energy consumption by NKA in L6 myotubes

Analysis of extracellular acidification rate (ECAR) and oxygen consumption rate (OCR) were analysed with Seahorse XFe24 Analyzer (Agilent Technologies). Rat L6 cells were seeded on Seahorse XF24 cell culture microplates (Agilent Technologies) at a density of 12,000 cells/well. After differentiation in MEMα with nucleosides (MEMα(+)) containing 2% (v/v) FBS for 7–10 days, the medium was replaced with MEMα without nucleosides (MEMα(-)) without FBS for the last 24 h. One hour before the experiment the medium was replaced with Seahorse XF DMEM medium (Agilent Technologies) supplemented with 10 mM glucose, 1 mM pyruvate and 2 mM glutamine. Cells were incubated for 1 h at 37 °C in normal atmosphere (no additional CO_2_) and then transferred into Seahorse and treated with monensin (0.5 µM, 5 µM and 25 µM) and/or ouabain (1 mM).

### Statistical analysis

The data are presented as mean values ± SD. Statistical analysis was performed with GraphPad Prism 6 (GraphPad Software). Significance (*P* < 0.05) was determined using one-way ANOVA followed by Bonferroni’s *post-hoc* test or t-test.

## Results

### Effect of glucose deprivation on the expression of NKA subunits in L6 myotubes

**Fig. 1:**
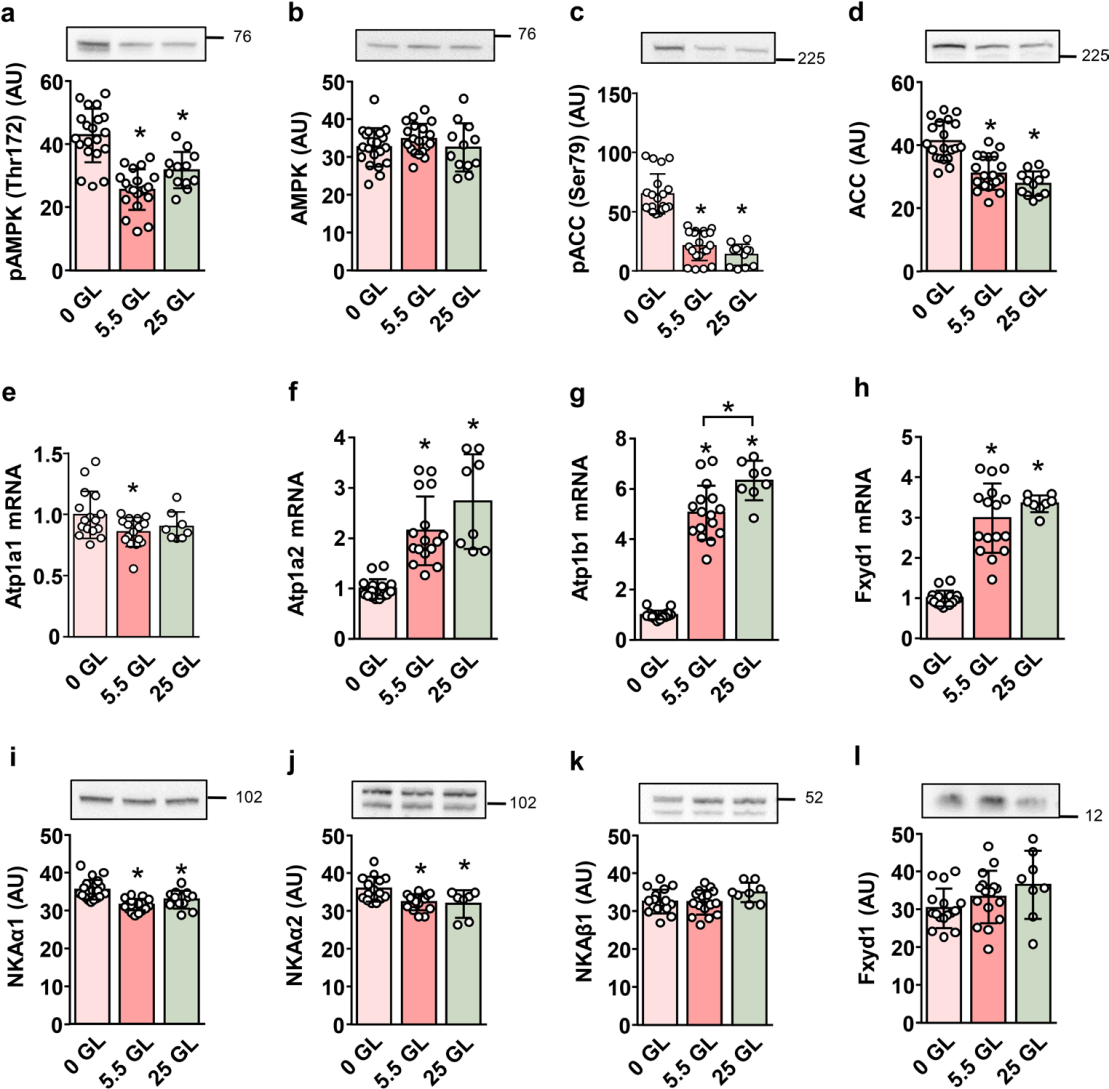
Effect of glucose deprivation on the expression of NKA subunits in L6 myotubes. L6 myoblasts were expanded and differentiated into myotubes in MEMα containing 5.5 mM (1 g/L) glucose as described in the Methods. Myotubes were cultured in serum-free DMEM containing 0, 5.5, or 25 mM glucose (GL) for the last 24 h of the experiment. Protein abundance of pAMPK (Thr172) (**a**), AMPK (**b**), pACC (Ser79) (**c**), ACC (**d**), and NKAα1 (**i**), NKAα2 (**j**), NKAβ1 (**k**), FXYD1 (**l**) was estimated by immunoblotting. Numbers next to the blots indicate molecular weight markers (kDa). Loading was assessed by Ponceau S staining. qPCR was used to estimate gene expression of NKAα1 (*Atp1a1*) (**e**), NKAα2 (*Atp1a2*) (**f**), NKAβ1 (*Atp1b1*) (**g**), and FXYD1 (*Fxyd1*) (**h**). Endogenous control: 18S rRNA. Results are means with SD (2–4 independent experiments, *n* = 7–20, *P < 0.05)

Rat L6 myotubes were cultured for 24 h in serum-free DMEM containing 0, 5.5 mM (1 g/L), or 25 mM (4.5 g/L) glucose (Fig. 1). Increased phosphorylation of the catalytic AMPKα subunit (Fig. 1a, b) at Thr172 and phosphorylation of the AMPK substrate acetyl-CoA carboxylase (ACC) at Ser79 (Fig. 1c, d) indicated that glucose deprivation led to energy stress in L6 myotubes. Glucose deprivation slightly increased the mRNA levels of NKAα1 (Fig. 1e), while it decreased those of NKAα2 (Fig. 1f), NKAβ1 (Fig. 1g), and FXYD1 (Fig. 1h). It should be noted, however, that, as estimated from the gene expression ratios, NKAα2 mRNA levels were less than 1% of NKAα1 mRNA levels, meaning that NKAα1 predominated under all conditions. Glucose deprivation increased protein levels of NKAα1 (Fig. 1i) and NKAα2 (Fig. 1j), with no effect on NKAβ1 (Fig. 1k) and FXYD1 (Fig. 1l).

### Energy consumption by NKA in L6 myotubes

**Fig. 2:**
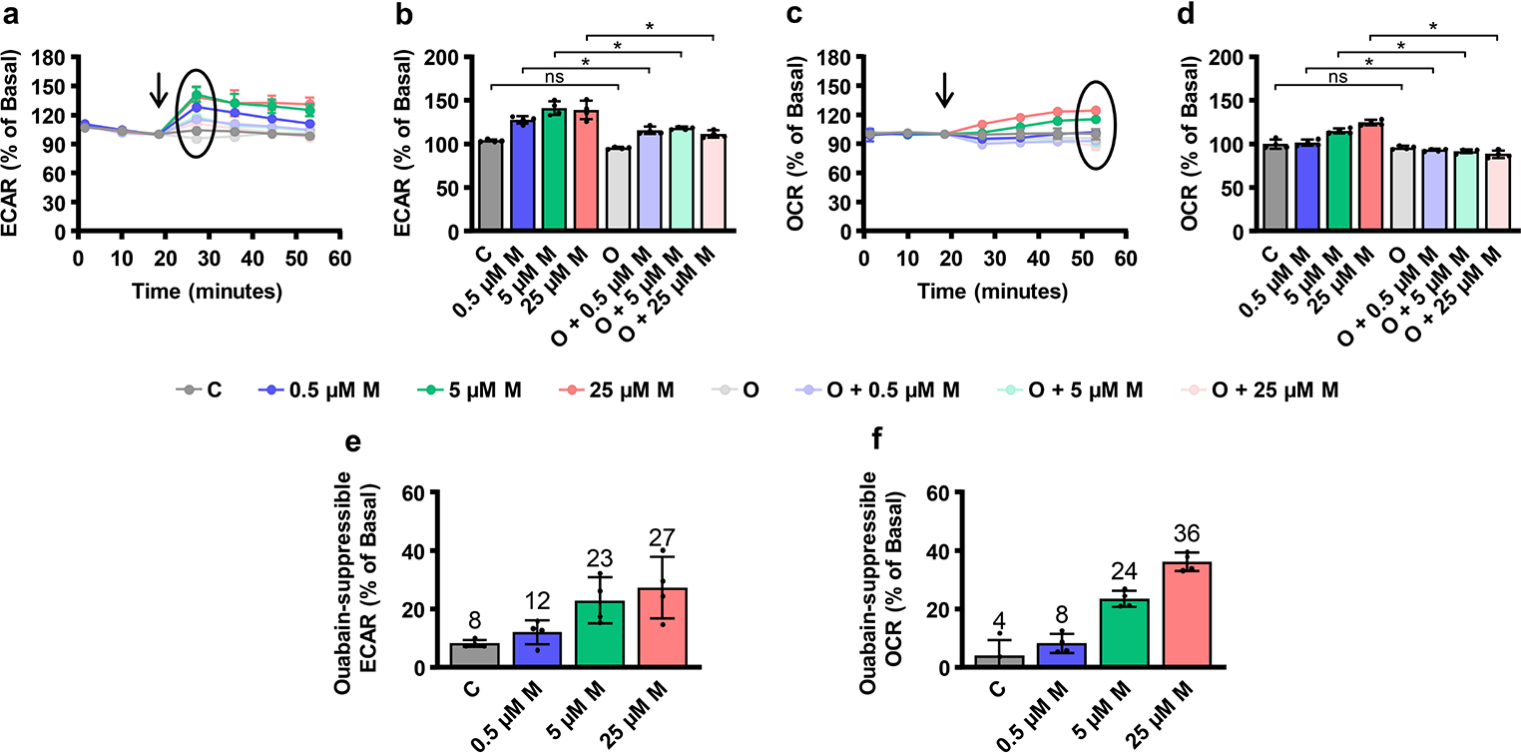
Energy consumption by NKA in L6 myotubes. Experiments were performed in the presence of 10 mM glucose. Real-time measurement of ECAR (**a**,**b**) and OCR (**c**,**d**) in L6 cells using Seahorse XF Analyzer. 3 measurements were taken before and 4 measurements were taken during treatment with vehicles, monensin (M; 0.5, 5 and 25 µM) and/or ouabain (O; 1 mM) (addition of compounds is indicated by an arrow). Bar graphs show 1st ECAR measurement and 4th OCR measurement after addition of compounds. Ouabain-suppressible ECAR (**e**) and OCR (**f**) were calculated as the difference between the cells treated with monensin + ouabain and cells treated with ouabain alone. Results are means with SD (2 independent experiments, *n* = 4, * *P* < 0.05)

To assess the energy consumption by NKA, OCR, and ECAR were measured in L6 myotubes (Fig. 2). Both were measured under basal conditions and in the presence of the NKA inhibitor ouabain, at a concentration (1 mM) chosen to completely inhibit NKA activity (Chibalin et al. 2012), and/or the Na^+^ ionophore monensin, at concentrations (0.5, 5, and 25 µM) that are known to activate NKA (Efendiev et al. 2002). In unstimulated L6 myotubes, ouabain did not significantly affect OCR and ECAR. Treatment with monensin increased OCR and ECAR in a concentration dependent-manner by up to ~ 25% and ~ 35%, respectively. Ouabain almost completely prevented effects of monensin, which shows that OCR and ECAR were increased due to activation of NKA.

### Effect of AMPK activators on the expression of NKA subunits in L6 myotubes

**Fig. 3:**
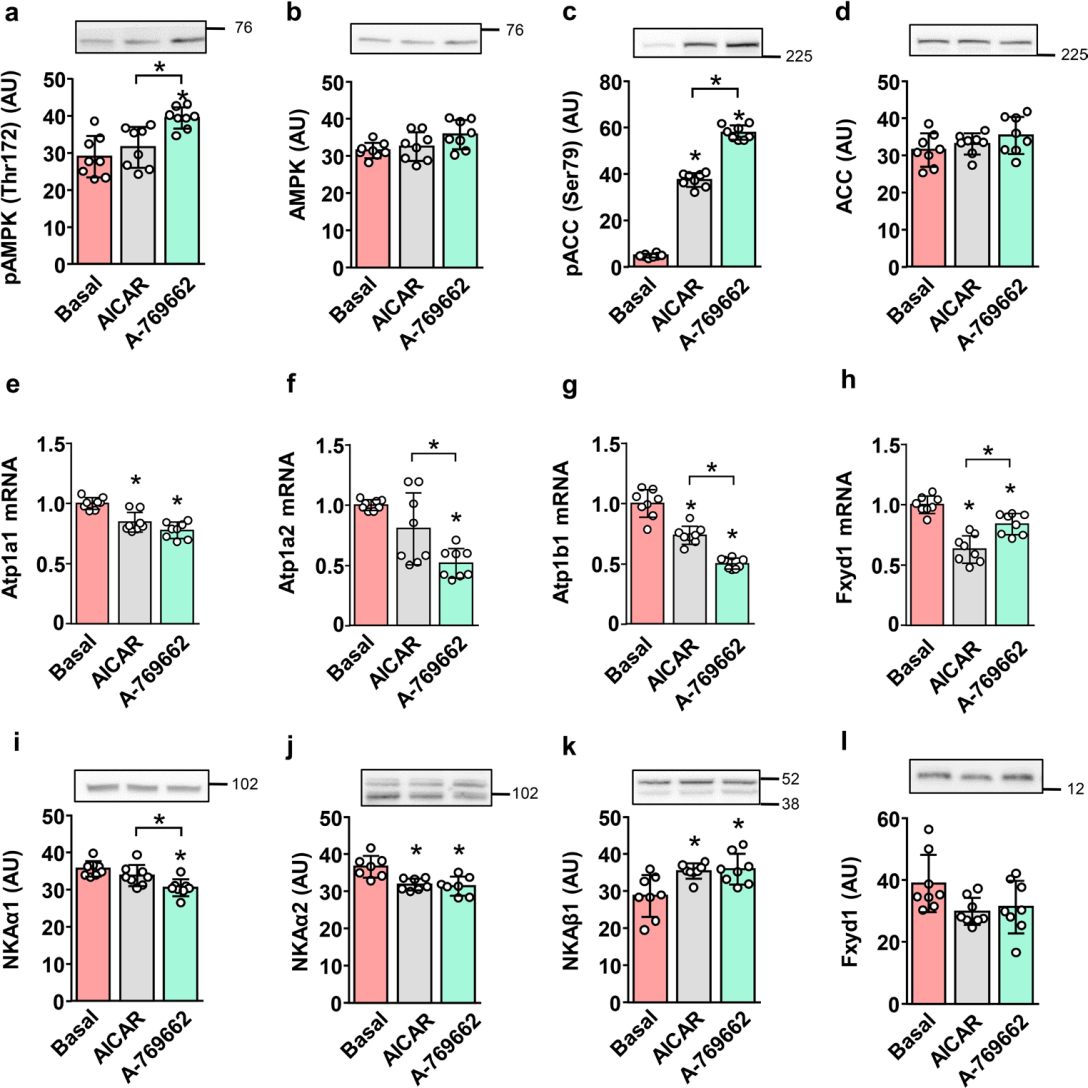
Effect of AMPK activators on the expression of NKA subunits in L6 myotubes. Experiments were performed in the presence of 5.5 mM (1 g/L) glucose. Myotubes, cultured in serum-free MEMα(-), were treated with AICAR (2 mM) and A-769662 (100 µM) for 24 h. Protein abundance of pAMPK (Thr172) (**a**), AMPK (**b**), pACC (Ser79) (**c**), ACC (**d**), NKAα1 (**i**), NKAα2 (**j**), NKAβ1 (**k**), and FXYD1 (**l**) was estimated by immunoblotting. Numbers next to the blots indicate molecular weight markers (kDa). Loading was assessed by Ponceau S staining. qPCR was used to estimate gene expression of NKAα1 (*Atp1a1*) (**e**), NKAα2 (*Atp1a2*) (**f**), NKAβ1 (*Atp1b1*) (**g**), and FXYD1 (*Fxyd1*) (**h**). Endogenous control: 18S rRNA. Results are means with SD (2 independent experiments, *n* = 7–8, *P < 0.05)

To determine whether activation of AMPK could explain the changes in NKA expression under glucose-deprived conditions, L6 myotubes were treated for 24 h with the AMPK activators AICAR (2 mM) and A-769662 (100 µM) in serum-free MEMα (Fig. 3). Phosphorylation of AMPKα (Fig. 3a, b) was increased by A-769662, while phosphorylation of ACC (Ser79) (Fig. 3c, d) was increased by AICAR and A-769662, indicating that AMPK was activated. Both activators decreased or tended to decrease the mRNA levels of NKAα1, NKAα2, NKAβ1, and FXYD1 (Fig. 3e-h). The reduction in mRNA levels was accompanied by a similar trend in protein levels of NKAα1, NKAα2 and FXYD1 (Fig. 3i, j, and l), although not all apparent differences reached statistical significance. The level of NKAβ1 protein (Fig. 3k) was increased by AICAR and A-769662, in contrast to its mRNA, which was reduced (Fig. 3g). These results demonstrate that effects of AMPK activators do not fully correspond to those observed under glucose-deprived conditions.

### Effect of gene silencing of AMPKα1 and AMPKα2 on the expression of the NKA subunits in L6 myotubes

**Fig. 4:**
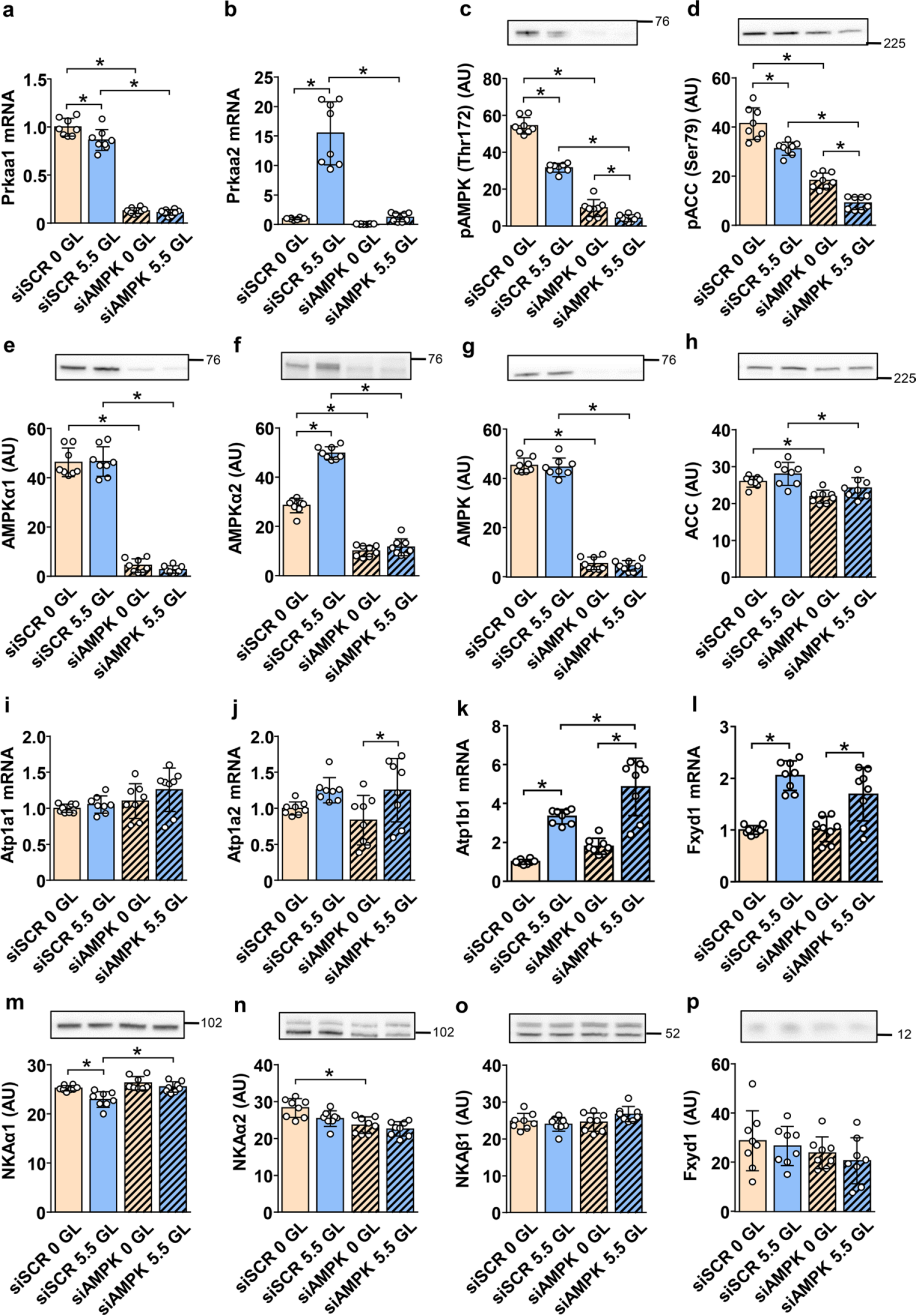
Effect of gene silencing of AMPKα1 and AMPKα2 on the expression of the NKA subunits in L6 myotubes. L6 myoblasts were expanded, differentiated into myotubes, and transfected with siRNA in the presence of 5.5 mM (1 g/L) glucose as described in the Methods. Control (siSCR) and AMPKα1/α2 (siAMPK) knock-down myotubes were grown in serum-free DMEM containing 0 or 5.5 mM glucose (GL) for the last 24 h of the experiment. qPCR was used to estimate gene expression of AMPKα1 (*Prkaa1*) (**a**), AMPKα2 (*Prkaa2*) (**b**), NKAα1 (*Atp1a1*) (**i**), NKAα2 (*Atp1a2*) (**j**), NKAβ1 (*Atp1b1*) (**k**), and FXYD1 (*Fxyd1*) (**l**). Endogenous control: 18S rRNA. Protein abundance of pAMPK (Thr172) (**c**), pACC (Ser79) (**d**), AMPKα1 (**e**), AMPKα2 (**f**), AMPK (**g**), ACC (**h**), NKAα1 (**m**), NKAα2 (**n**), NKAβ1 (**o**), and FXYD1 (**p**) was estimated by immunoblotting. Numbers next to the blots indicate molecular weight markers (kDa). Loading was assessed by Ponceau S staining. Results are presented as means with SD (2 independent experiments, *n* = 8, *P < 0.05)

To determine whether effects of glucose deprivation are dependent on AMPK activation, the catalytic AMPKα1 and AMPKα2 subunits were knocked-down by transfection of L6 myotubes with siRNA, which were then cultured in DMEM containing 0, 5.5, or 25 mM glucose (Fig. 4). The mRNA levels of AMPKα1 (*Prkaa1*) and AMPKα2 (*Prkaa2*) were reduced in the knock-down myotubes (Fig. 4a, b), as was the abundance of phosphorylated AMPKα (Fig. 4c), total AMPKα (Fig. 4g), and phosphorylated ACC (Fig. 4d). In control myotubes, AMPKα2 expression was strongly dependent on the presence of glucose (Fig. 4b, f). Gene silencing of AMPKα1 and AMPKα2 reduced also total ACC levels (Fig. 4h). Despite the decrease in AMPKα levels, glucose deprivation increased the phosphorylation of AMPK (Fig. 4c, g) and ACC (Fig. 4d, h), demonstrating that the knock-down L6 myotubes have low, but still significant residual AMPK activity.

In the presence of glucose, gene silencing of AMPKα1 and AMPKα2 had no effect on NKAα1 (Fig. 4i), NKAα2 (Fig. 4j), and FXYD1 (Fig. 4l) mRNA levels, while it increased NKAβ1 levels (Fig. 4k). Gene silencing also increased protein levels of NKAα1 (Fig. 4m), while it decreased those of NKAα2 (Fig. 4n). It had no effect on the levels of NKAβ1 (Fig. 4o) and FXYD1 (Fig. 4p). Glucose deprivation decreased or tended to decrease mRNA levels of NKAα2 (Fig. 4j), NKAβ1 (Fig. 4k), and FXYD1 (Fig. 4l) in the control and knock-down myotubes, with no effect on mRNA levels of NKAα1 (Fig. 4i). In the control, but not in the knock-down myotubes, glucose deprivation increased NKAα1 protein (Fig. 4m). The protein levels of NKAβ1 (Fig. 4o) and FXYD1 (Fig. 4p) were similar under all conditions.

### Effect of AMPK activators in the AMPKα1/α2 knock-down L6 myotubes

**Fig. 5:**
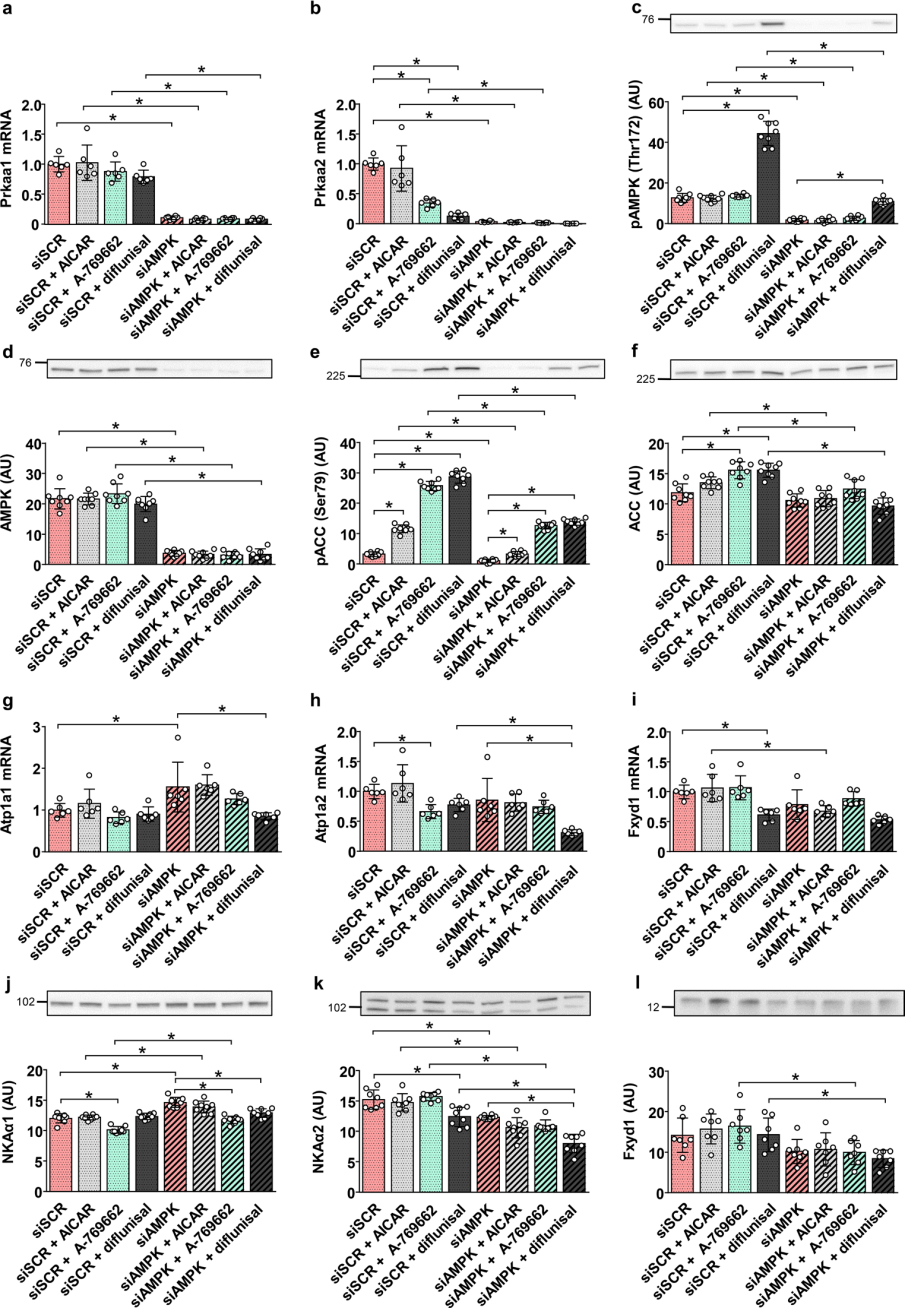
Effect of AMPK activators in the AMPKα1/α2 knock-down L6 myotubes. Experiments were performed in the presence of 5.5 mM (1 g/L) glucose. Control (siSCR) and AMPKα1/α2 (siAMPK) knock-down myotubes, cultured in in MEMα (-), were treated with AICAR (2 mM), A-769662 (100 µM), or diflunisal (200 µM) for 24 h. qPCR was used to estimate gene expression of AMPKα1 (*Prkaa1*) (**a**), AMPKα2 (*Prkaa2*) (**b**), NKAα1 (*Atp1a1*) (**g**), NKAα2 (*Atp1a2*) (**h**), and FXYD1 (*Fxyd1*) (**i**). Endogenous control: 18S rRNA. Protein abundance of pAMPK (Thr172) (**c**), AMPK (**d**), pACC (Ser79) (**e**), ACC (**f**), NKAα1 (**j**), NKAα2 (**k**), and FXYD1 (**l**) was estimated by immunoblotting. Numbers next to the blots indicate molecular weight markers (kDa). Loading was assessed by Ponceau S staining. Results are means with SD (2 independent experiments, *n* = 7–8 for immunoblotting, *n* = 6 for qPCR, *P < 0.05)

To further investigate the effects of AMPK on NKA expression, the AMPKα1/α2 knock-down myotubes were treated with the AMPK activators AICAR, A-769662, and diflunisal. Although gene silencing effectively suppressed the expression of AMPKα1 and AMPKα2 mRNA (Fig. 5a, b) and the level of phosphorylated (Fig. 5c) and total AMPKα (Fig. 5d), A-769662 and diflunisal were still able to activate AMPK and increase the phosphorylation of ACC (Fig. 5e). The levels of total ACC were again lower in the knock-down myotubes than in controls (Fig. 5f).

Gene silencing of AMPKα1 and AMPKα2 increased NKAα1 mRNA (Fig. 5g) and protein (Fig. 5j) levels, while it reduced NKAα2 (Fig. 5k) protein levels and FXYD1 (Fig. 5i) mRNA levels. AICAR, which was a weaker AMPK activator than A-769662 and diflunisal, did not affect the expression of any of the NKA subunits. In contrast, diflunisal, which was the strongest AMPK activator (Fig. 5c, e), reduced mRNA and protein levels of NKAα1 and NKAα2 (Fig. 5g, h, j, k), while it also exerted a suppressive effect on the expression of FXYD1 (Fig. 5i, l). Of the three AMPK activators, A-769662 was the most effective suppressor of NKAα1 protein levels (Fig. 5j).

### Effect of glucose on the expression of NKA subunits in primary human myotubes

**Fig. 6:**
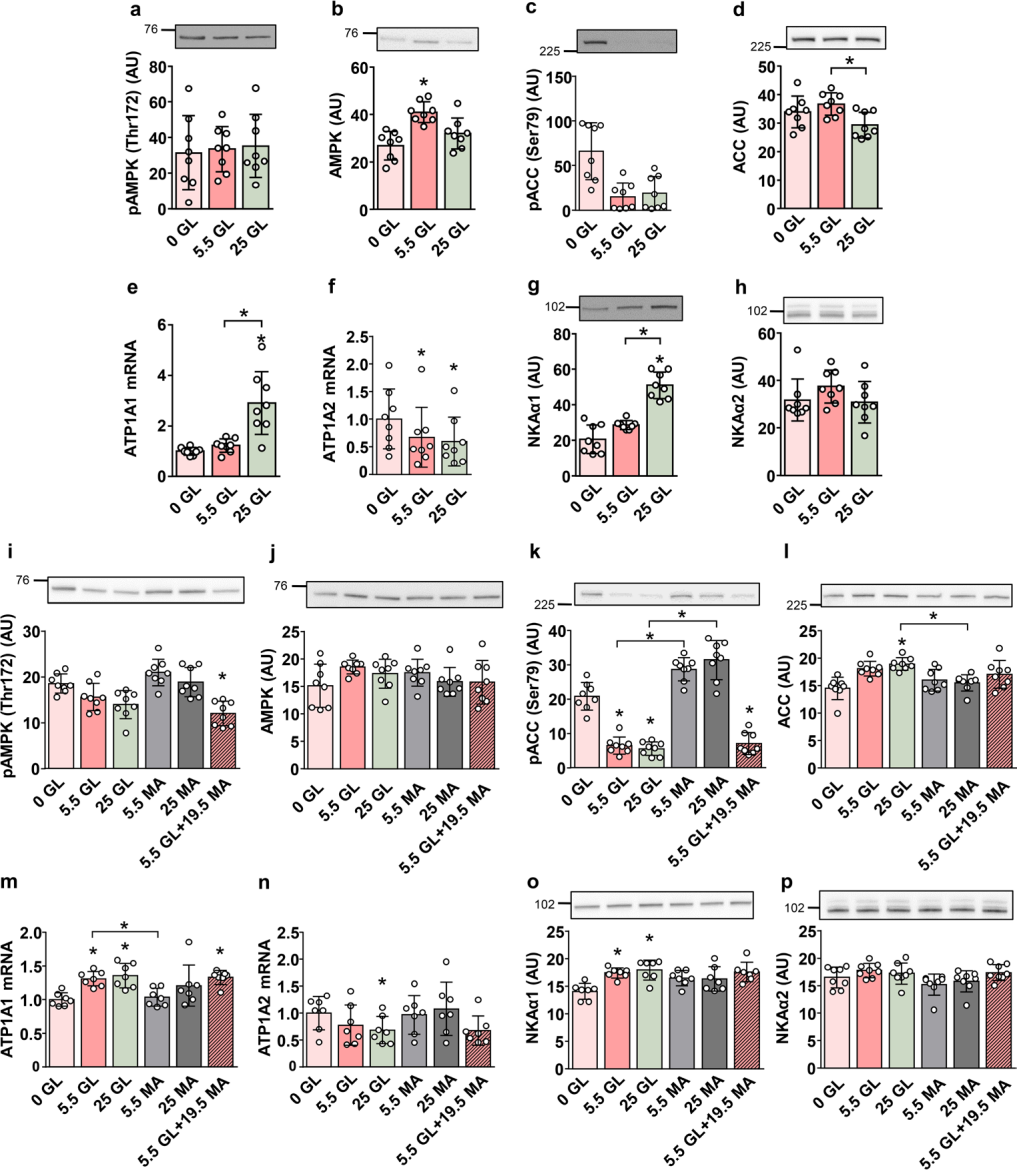
Effect of glucose deprivation and mannitol on the expression of NKA in primary human myotubes. Myoblasts were expanded and differentiated into myotubes in DMEM containing 5.5 mM (1 g/L) glucose as described in the Methods. (**a**-**h**) Myotubes were cultured in DMEM containing 0, 5.5, and 25 mM glucose (GL) for 24 h. (**i**-**p**) Myotubes were cultured (24 h) in DMEM containing 0, 5.5, and 25 mM glucose (GL) and/or 5.5, 19.5, and 25 mM mannitol (MA) as indicated. Protein abundance of pAMPK (Thr172) (**a**, **i**), AMPK (**b**, **j**), pACC (Ser79) (**c**, **k**), ACC (**d**, **l**), NKAα1 (**g**, **o**) and NKAα2 (**h**, **p**) was estimated by immunoblotting. Numbers next to the blots indicate molecular weight markers (kDa). Loading was assessed by Ponceau S staining. qPCR was used to estimate gene expression of NKAα1 (*ATP1A1*) (**e**, **m**) and NKAα2 (*ATP1A2*) (**f**, **n**). Endogenous control: 18S rRNA. Results are means with SD (2 independent experiments, *n* = 8, *P < 0.05)

Results in L6 myotubes indicated that glucose and AMPK regulate NKAα subunits in an isoform-specific manner. To investigate whether responses of NKAα1 and NKAα2 in human cells are similar, primary human myotubes were cultured in serum-free DMEM containing 0, 5.5, or 25 mM glucose for 24 h (Fig. 6). Glucose deprivation did not alter the phosphorylation of AMPK (Fig. 6a, b), while apparently increasing the phosphorylation of ACC (Fig. 6c, d), although the difference did not reach the level of statistical significance. NKAα1 mRNA levels increased in parallel with increasing glucose concentrations (Fig. 6e), while those of NKAα2 decreased (Fig. 6f). In contrast to L6 myotubes, human myotubes expressed NKAα1 (~ 55% of transcripts) and NKAα2 (~ 45% of transcripts) at approximately equal levels under basal conditions. Glucose promoted the protein expression of NKAα1 (Fig. 6g), with no effect on NKAα2 (Fig. 6h).

To determine whether the observed effects were due to altered glucose concentrations or changes in osmolarity, primary human myotubes were exposed to different concentrations of glucose or mannitol (Fig. 6i-p). As estimated from the phosphorylation of ACC (Fig. 6k, l), glucose deprivation activated AMPK, although the phosphorylation of AMPK was not altered (Fig. 6i, j). NKAα1 mRNA and protein levels were upregulated in the presence of glucose, but not in the presence of mannitol alone (Fig. 6m, o). NKAα2 mRNA was downregulated by increasing glucose concentrations, but not when mannitol was used instead of glucose (Fig. 6n). There was no change in NKAα2 protein (Fig. 6p).

### Effect of gene silencing of AMPKα1/α2 on the expression of NKAα1 and NKAα2 in primary human myotubes

**Fig. 7:**
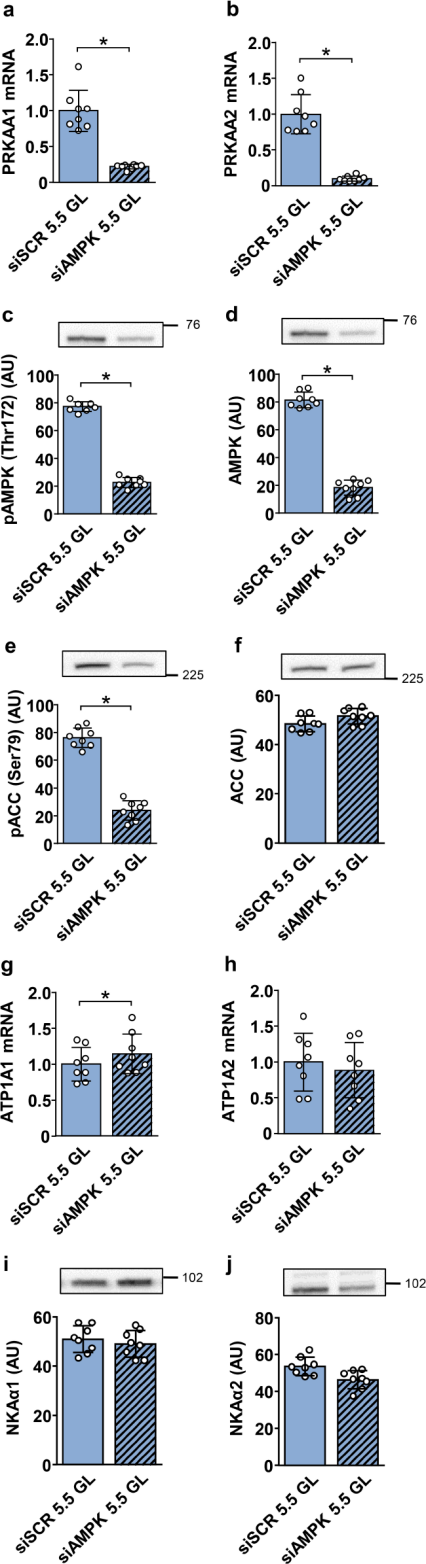
Effect of AMPKα1/α2 knock-down in primary human myotubes. Myoblasts were expanded, differentiated into myotubes, and transfected with siRNA in the presence of 5.5 mM (1 g/L) glucose as described in the Methods. Control and AMPKα1/α2 knock-down myotubes were cultured in serum-free DMEM (5.5 mM glucose) for 24 h. qPCR was used to estimate gene expression of AMPKα1 (*PRKAA1*) (**a**), AMPKα2 (*PRKAA2*) (**b**), NKAα1 (*ATP1A1*) (**g**), and NKAα2 (*ATP1A2*) (**h**). Endogenous control: 18S rRNA. Protein abundance of pAMPK (Thr172) (**c**), AMPK (**d**), pACC (Ser79) (**e**), ACC (**f**), NKAα1 (**i**), and NKAα2 (**j**) was estimated by immunoblotting. Numbers next to the blots indicate molecular weight of marker (kDa). Loading was assessed by Ponceau S staining. Results are means with SD (2 independent experiments, *n* = 8,*P < 0.05)

The catalytic AMPKα1 and AMPKα2 subunits were knocked-down with siRNA (Fig. 7a, b) and cultured in DMEM containing 0 or 5.5 mM glucose. However, AMPKα1/α2 knock-down cells were not viable in glucose-free DMEM, so we only show the results obtained under 5.5 mM glucose. The knock-down myotubes had lower levels of phosphorylated AMPK (Fig. 7c, d) and ACC (Fig. 7e, f). NKAα1 mRNA (Fig. 7g) was slightly increased in the knock-down myotubes, its proteins levels (Fig. 7i) and the expression of NKAα2 (Fig. 7h, j) were not changed.

## Discussion

Our study shows that glucose deprivation and AMPK affect the expression of NKA subunits in cultured myotubes. It also shows that effects of glucose deprivation only partially correspond to those of AMPK activation, suggesting that AMPK does not mediate all effects of energy stress on NKA expression. These results provide a proof of principle that energy stress and AMPK can affect NKA expression in myotubes. However, responses were not conserved between L6 myotubes and primary human myotubes, which suggests that coupling between AMPK and regulation of NKA expression depends on skeletal muscle cell model and/or species.

Glucose deprivation activated AMPK and increased protein levels of NKAα1 and NKAα2 in L6 myotubes, which shows that AMPK is not only able to activate ATP consumers such as NKA in L6 myotubes (Benziane et al. 2012) and sarcoplasmic/endoplasmic reticulum ATPase (SERCA) in smooth muscle cells (Schneider et al. 2015), but also to increase their abundance. Upregulation of NKAα1 protein was observed also in human skeletal muscle after exercise with blood flow restriction (Christiansen et al. 2019), which causes sufficient energy stress to activate AMPK (Christiansen et al. 2018), consistent with the notion that activation of AMPK is not always associated with suppression of energy consumers. In contrast, human primary myotubes responded to glucose deprivation with downregulation of protein expression of NKAα1, implying that energy stress-induced upregulation of NKAα1 is not a general property of skeletal muscle cells. While the molecular basis of the different responses in L6 and human myotubes remains to be elucidated, it is important to note that protein expression of NKAα1 protein was altered under glucose deprivation in both types of myotubes, consistent with the idea that energy stress can modulate the abundance of NKA subunits in skeletal muscle.

In L6 myotubes, gene silencing of AMPKα1/α2 increased protein levels of NKAα1, while decreasing those of NKAα2, implicating a role for AMPK in regulation of NKA content in skeletal muscle. Excepting a minor increase in NKAα1 mRNA levels, these effects were not observed after knocking-down AMPKα1/α2 in human myotubes, meaning it is not possible to make any generalizations about the role of AMPK in regulation of NKA. However, focusing on L6 myotubes, it is interesting to compare effects of gene silencing of AMPKα1/α2 and glucose deprivation. While gene silencing experiments suggest that AMPK is a negative regulator of NKAα1 and a positive regulator of NKAα2, the abundance of both isoforms was increased under glucose-deprived conditions concurrently with AMPK activation. When considered together, these results suggest that under glucose-deprived conditions upregulation of NKAα1 occurred despite activation of AMPK, whereas upregulation of NKAα2 may have occurred because of AMPK activation. These results also suggest that AMPK does not mediate all effects of energy stress on NKA expression.

Downregulation of NKAα1 in glucose-deprived human myotubes seems to be consistent with the role of AMPK as the maintainer of energy balance, while upregulation of NKAα2 in glucose-deprived L6 myotubes seems to oppose it. However, changes in NKA protein levels should not be interpreted as evidence that energy usage by NKA was altered. Importantly, we did not measure activity of NKA, so functional consequences of the observed upregulation are not known. Moreover, as evident from our Seahorse experiments in L6 myotubes, the ouabain-suppressible OCR and ECAR in L6 myotubes were ~ 4–8% of the total, which increased by ~ 24% and ~ 38%, respectively, during exposure to the highest concentration of Na^+^-ionophore monensin. In the monensin-treated myotubes, the ouabain-suppressible component of OCR and ECAR were 36% and 27%, respectively. These results show, firstly, that NKA uses only a minor fraction of energy in unstimulated L6 myotubes, consistent with the estimate that NKA in skeletal muscle uses less than 10% of energy (Biron et al. 1979; Rolfe and Brown 1997). Secondly, our results show that L6 myotubes have a huge reserve capacity for Na^+^-K^+^ transport, which is revealed only when NKA is stimulated. This is again consistent with data from isolated skeletal muscle, which show that electrical stimulation can activate NKA up to 18-fold (Nielsen and Clausen 1997).

None of the AMPK activators increased protein expression of NKAα1 or NKAα2 in L6 myotubes. Indeed, despite some variability between different compounds and experimental conditions, AMPK activators seemed to exert a suppressive effect on NKAα mRNA and/or protein expression. On the one hand these results are consistent with results of gene silencing of AMPKα1/α2, which indicate that AMPK is a negative regulator of NKAα1. They are also consistent with the notion that glucose deprivation increased NKAα1 protein expression in an AMPK-independent manner. However, on the other hand, gene silencing experiments indicated that AMPK is a positive regulator of NKAα2, based on which upregulation of NKAα2 would be expected during treatment with AMPK activators. One explanation for the observed discrepancy could be that AMPK only has a permissive effect on NKAα2 expression under basal conditions, but does not positively regulate NKA expression when activated. It is also possible that AMPK activation under energy-deprived conditions, such as in glucose deprivation experiments, has a different effect on NKA expression compared with pharmacological AMPK activation under energy-replete conditions, such as in experiments with AMPK activators. Finally, it is important to consider that AMPK activation, as estimated by the phosphorylation of ACC, under glucose-deprived conditions was relatively modest compared with treatment with AMPK activators, which could be another reason for seemingly inconsistent results.

Effects of pharmacological AMPK activators on NKA expression were not always the same. As estimated from the phosphorylation of ACC, AICAR was the least potent and diflunisal was the most potent AMPK activator, which could explain some of the differences between them. Moreover, although pharmacological agents are a useful and widely used approach to study the effects of AMPK, caution is always required when interpreting their effects on NKA due to their potential off-target effects (Pirkmajer et al. 2021). For instance, A-769662 binds to and inhibits NKAα, leading to its internalization (Benziane et al. 2009). A reduction in the abundance of NKAα1 in L6 myotubes treated with A-769662 could therefore be interpreted as an AMPK-independent event. Conversely, AICAR and diflunisal, a particularly potent AMPK activator, did not reduce the protein abundance of NKAα1, suggesting that AMPK activation per se did not suppress NKAα1 protein levels under these circumstances. Nevertheless, suppressive effect of AMPK on NKA expression cannot be completely excluded since both AICAR and A-769662 reduced NKAα1 mRNA levels.

Three important caveats need to be considered when interpreting results of AMPKα1/α2 gene silencing experiments. Firstly, despite markedly reducing AMPKα1/α2 mRNA and protein levels, gene silencing did not eliminate AMPK activity. Indeed, the remaining AMPK was activatable, and despite its low levels, was able to increase phosphorylation of ACC. It should therefore be assumed that AMPK activation, although significantly blunted, could have contributed to responses under glucose-deprived conditions. Another caveat is that some of the responses to AMPK activators were not completely consistent between experiments presented in Figs. 2 and 4. The most likely explanation is that this discrepancy is due to the gene silencing protocol to which myotubes were subjected in order to knock-down AMPKα1/α2. For instance, transfection reagent, which is needed to transfect myotubes with siRNA, could have affected membrane properties and expression of NKA. Thirdly, it needs to be emphasized that alterations in mRNA and protein levels of NKA subunits were frequently not congruous, meaning that the site of NKA regulation by energy stress (glucose deprivation) and AMPK is not clear. Divergent mRNA and protein responses of NKA subunits were observed before (Galuska et al. 2009) and may simply reflect different temporal patterns of transcriptional and posttranscriptional events. Alternatively, they may indicate existence of distinct transcriptional and posttranscriptional regulatory mechanisms.

Despite the caveats there are some interesting observations regarding the available in vivo data. In our study, glucose deprivation and AMPK activators suppressed FXYD1 mRNA expression, while chronic treatment with AICAR decreased the abundance of total FXYD1 in mouse skeletal muscle (Ingwersen et al. 2011). However, FXYD1 levels in L6 myotubes were not affected (or even tended to be suppressed) by gene silencing of AMPKα1/α2, while FXYD1 was upregulated in skeletal muscle of AMPK kinase-dead mice (Ingwersen et al. 2011). Furthermore, although AICAR suppressed NKAα2 and increased NKAβ1 protein levels in L6 myotubes, there was no change in NKAα and NKAβ subunits in the skeletal muscle of AICAR-treated mice (Ingwersen et al. 2011). Similarly, knock-down of AMPKα1/α2 in L6 myotubes upregulated NKAα1 and downregulated NKAα2, whereas these subunits remained unchanged in skeletal muscle of AMPK kinase-dead mice (Ingwersen et al. 2011). These differences indicate that the molecular mechanisms linking AMPK to the regulation of NKA subunits, including FXYD1, are not conserved in cultured myotubes. However, it is also possible that a 24-hour exposure to AICAR or a short-term partial loss of AMPK function in the L6 myotubes are not sufficient to induce the same adaptations as a 27-day treatment with AICAR in wild-type mice or a complete and permanent loss of AMPK activity in the AMPK kinase-dead mice.

Upregulation of NKAα1 mRNA and protein in primary human myotubes shows that availability of glucose can modulate expression of NKA subunits in cultured myotubes. An increase in NKAα1 mRNA was observed also in vascular smooth muscle cells exposed to high glucose concentrations (Muto et al. 1998) and in choroid plexus of diabetic rats (Egleton et al. 2003). Furthermore, sedentary rats fed a high fat diet displayed insulin resistance, dysregulated glucose homeostasis, as well as increased levels of NKAα1 mRNA and protein in *gastrocnemius* muscle (Galuska et al. 2009). Importantly, exposure to hyperosmotic media also increased NKAα1 mRNA levels in vascular smooth muscle cells (Muto et al. 1998), indicating that effects of glucose were not specific in these cells. Conversely, the expression of NKA subunits was unaltered in primary human myotubes exposed to mannitol. Although osmotic effects of glucose cannot be completely ruled out, this result suggests that the addition or withdrawal of glucose was the main factor leading to changes in NKA subunit expression in our study.

Regulation of gene expression is a major mechanism by which various stimuli, including exercise and hormones, determine NKA content in skeletal muscle and thereby its maximal capacity to perform ion transport (Nielsen and Clausen 1997; Clausen 2003; Pirkmajer and Chibalin 2016). However, NKA activity during high frequency electrical stimulation of skeletal muscle is still well below the theoretical maximum pumping capacity (Nielsen and Clausen 1997), indicating that not all pumps are available to perform ion transport. Therefore, activity of individual pump units and their distribution between the membrane and the intracellular compartment also need to be considered (Tsakiridis et al. 1996; Benziane and Chibalin 2008). For instance, activation of AMPK was shown to decrease phosphorylation of NKAα1, while increasing its membrane abundance and NKA activity in L6 myotubes (Benziane et al. 2012). In contrast to AMPK, high glucose concentrations did not alter distribution of NKAα in isolated skeletal muscle (Chibalin et al. 2001), while they increased phosphorylation of NKAα and reduced NKA activity in isolated soleus muscle (Chibalin et al. 2001) and isolated murine pancreatic islets (Owada et al. 1999). In contrast to in vitro results, oral glucose supplements administered during prolonged exercise result in a transient increase in maximal NKA activity in contracting human *vastus lateralis* muscle (Green et al. 2007). Clearly, while the purpose of our study was to establish whether energy stress, induced by glucose deprivation, and/or AMPK modulate expression of NKA in cultured myotubes, it would be also important to examine phosphorylation and subcellular distribution of NKA.

Primary human myotubes responded differently to altered glucose concentrations than L6 myotubes, αα which may reflect well-known species-specific functional properties of human and rat NKAα subunits. For example, while the rodent NKAα1 subunit is much less sensitive to cardiotonic glycosides such as ouabain than the human NKAα1 (Wang et al. 2001; Benziane et al. 2009; Chibalin et al. 2012), it is much more sensitive to inhibition by A-769662 (Benziane et al. 2009). Nevertheless, putative species-specific differences in functional properties of NKAα1 cannot provide a complete explanation for the observed differences, as NKAα1 mRNA was also markedly upregulated in rat vascular smooth muscle cells during exposure to 25 mM glucose (Muto et al. 1998), which is consistent with what we observed in primary human myotubes. It is therefore possible that different responses, which we observed in L6 and human myotubes, are simply the consequence of using two distinct experimental models, which differ in several important respects (Abdelmoez et al. 2020). Indeed, while primary human myotubes expressed similar proportions of NKAα1 and NKAα2, L6 myotubes expressed almost exclusively NKAα1, consistent with our previous observations (Pirkmajer et al. 2020; Jan et al. 2021). Altered expression of NKAα2 is therefore probably to have more functional consequences for primary human myotubes than for L6 myotubes.

## Conclusions

(1) Energy stress, induced by glucose deprivation, modulated protein expression of NKAα1 and α2 subunits in rat L6 and primary human myotubes. Divergent responses to energy stress in L6 myotubes (increased content of NKAα1 and NKAα2 protein) and human primary myotubes (decreased content of NKAα1 protein) suggest that the coupling between energy stress and the regulation of NKA expression is cell type-specific rather than an intrinsic property of a particular NKA subunit isoform.

(2) Results of gene silencing of AMPKα1/α2 in L6 myotubes demonstrate that under basal conditions AMPK suppresses protein expression of NKAα1, while promoting that of NKAα2. However, AMPK appears to be a less potent regulator of NKA expression in human myotubes, as only a minor upregulation of NKAα1 mRNA was observed after gene silencing of AMPKα1/α2.

(3) Changes in NKA expression, which occurred in response to pharmacological AMPK activation, only partially mimicked those that occurred in response to glucose deprivation, indicating that AMPK does not mediate all effects of energy stress on NKA expression.

### Electronic supplementary material

Below is the link to the electronic supplementary material.

Supplementary Material 1

Supplementary Material 2

## Data Availability

Raw data are available in the supplement.
