# Supplementary material for: AMPK and glucose deprivation exert an isoform-specific effect on the expression of Na+,K+-ATPase subunits in cultured myotubes
Source: J Muscle Res Cell Motil. 2024 May 6;45(3):139–54. doi: 10.1007/s10974-024-09673-9 (PMC11316707; doi:10.1007/s10974-024-09673-9)
Supplement: Supplementary file 1 — Supplementary Material 1 [file 10974_2024_9673_MOESM1_ESM.pdf]

RAW DATA of IMMUNOBLOTTING
Figure1, 3-7

AMPK and glucose deprivation exert an isoform-specific effect on the expression of Na^+,K^+-ATPase subunits in cultured myotubes

Figure 1

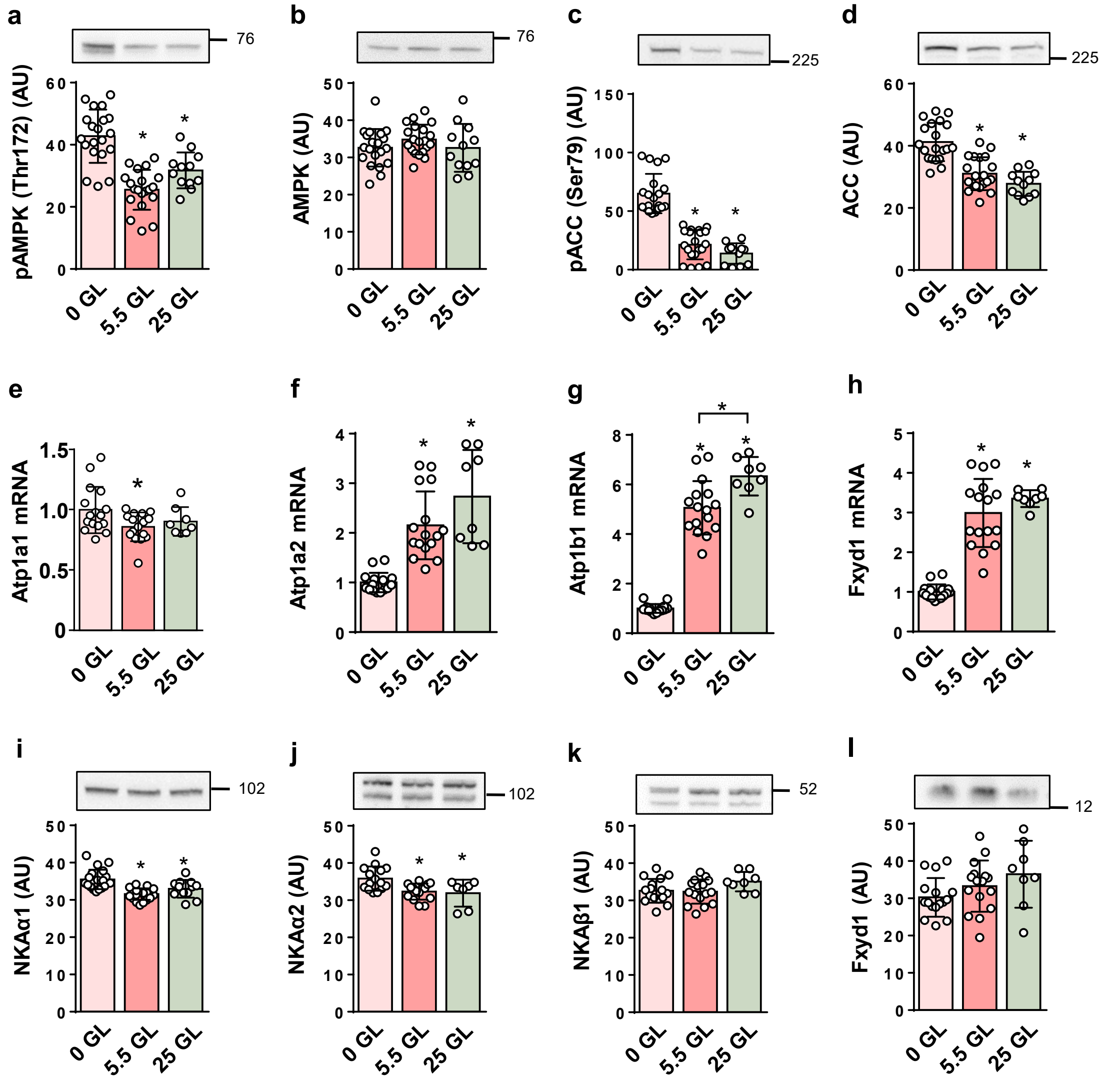

a
76
pAMPK (Thr172) (AU)
0 GL
5.5 GL
25 GL
b
76
AMPK (AU)
c
225
pACC (Ser79) (AU)
d
225
ACC (AU)
e
Atp1a1 mRNA
f
Atp1a2 mRNA
g
Atp1b1 mRNA
h
Fxyd1 mRNA
i
102
NKAα1 (AU)
j
102
NKAα2 (AU)
k
52
NKAβ1 (AU)
l
12
Fxyd1 (AU)

Figure 1

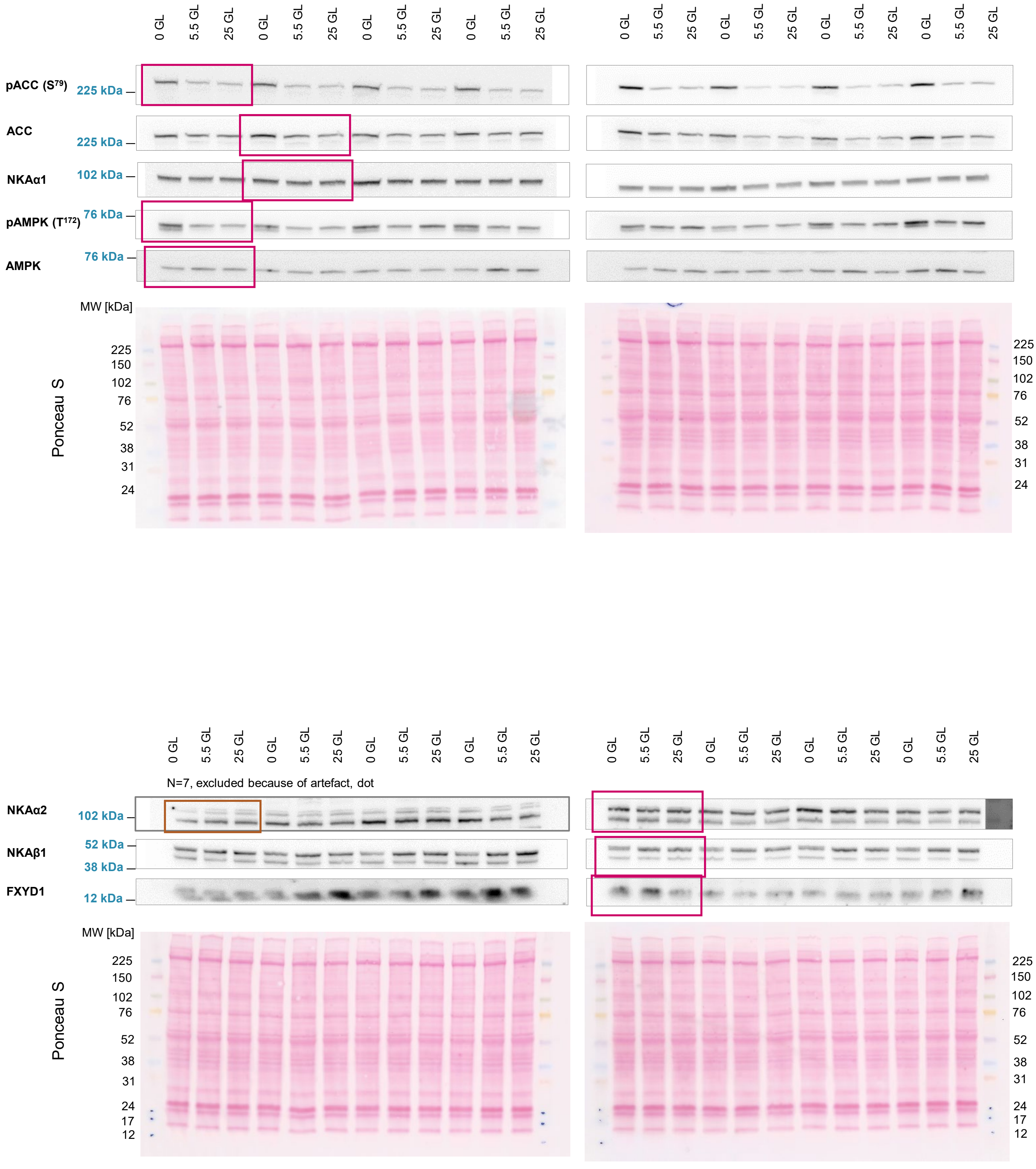

The frame shows the blots that are presented in the figure.

Figure 1

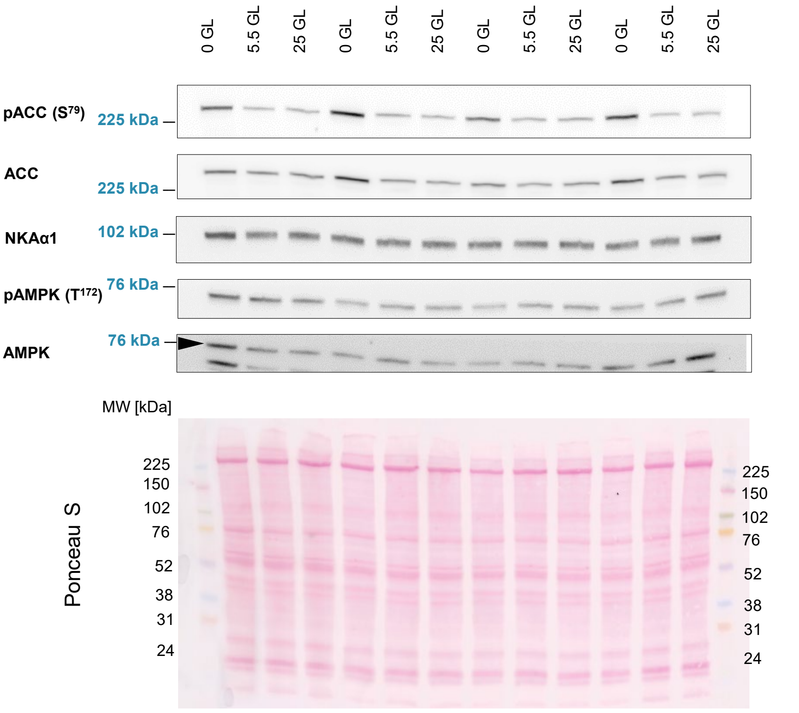

0 GL
5.5 GL
25 GL
0 GL
5.5 GL
25 GL
0 GL
5.5 GL
25 GL
0 GL
5.5 GL
25 GL
pACC (S79)
225 kDa
ACC
225 kDa
NKAα1
102 kDa
pAMPK (T172)
76 kDa
AMPK
76 kDa
MW [kDa]
Ponceau S
225
150
102
76
52
38
31
24
225
150
102
76
52
38
31
24

Figure 1

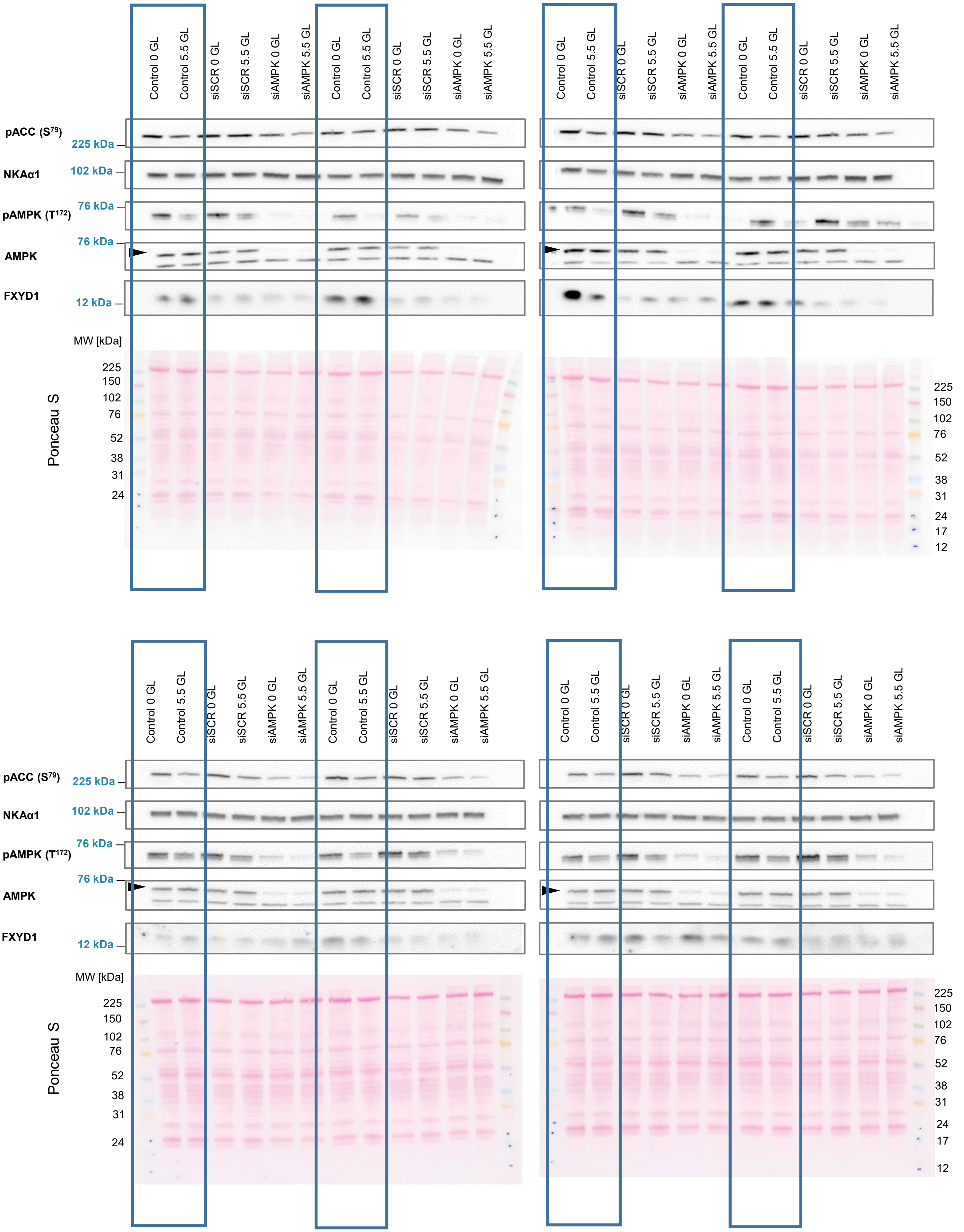

The blue frame shows analyzed bands from this experiment

Other treatments are shown again and analyzed in Figure 4

Figure 1

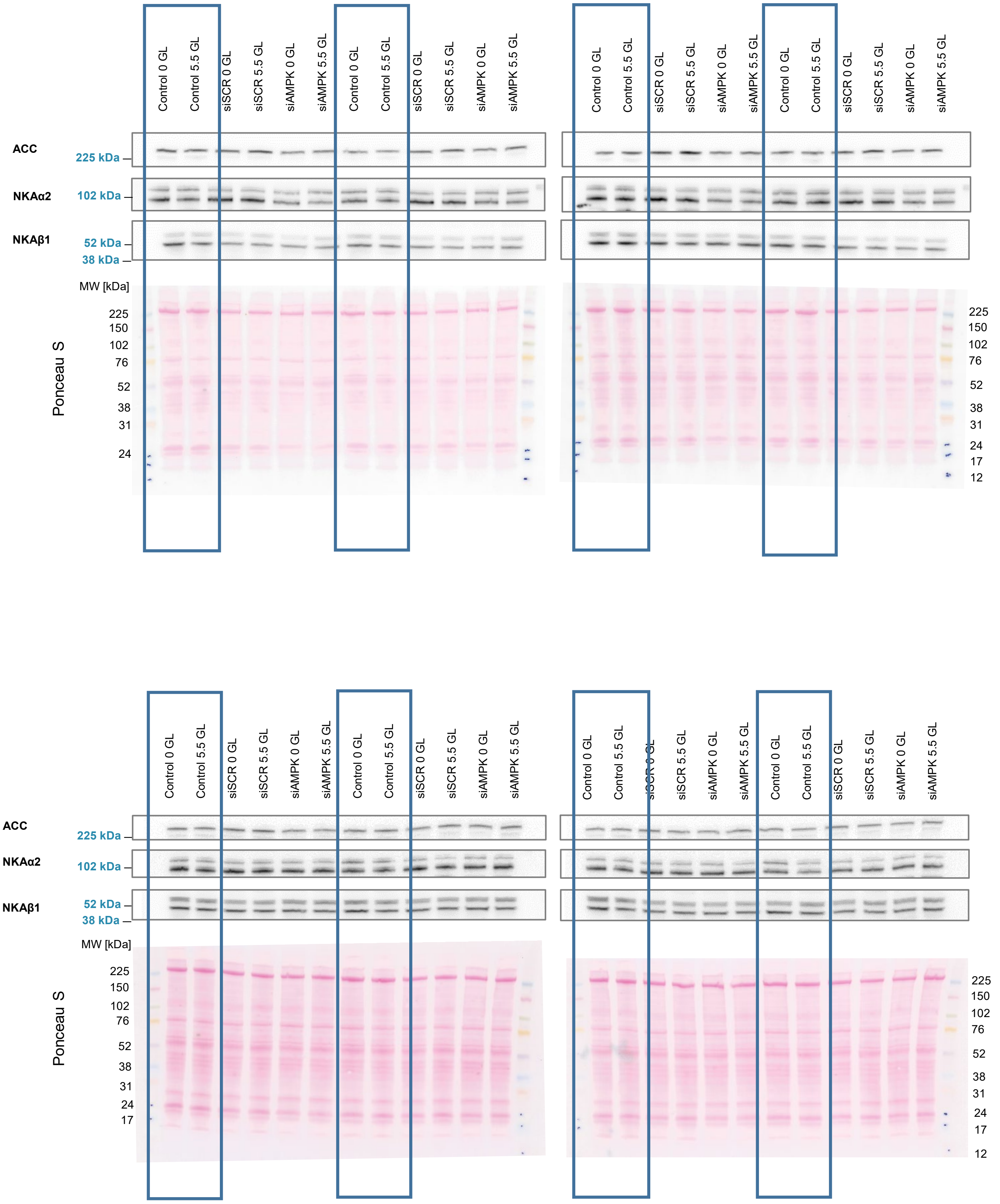

The blue frame shows analyzed bands from this experiment

Other treatments are shown again and analyzed in Figure 4

Figure 3

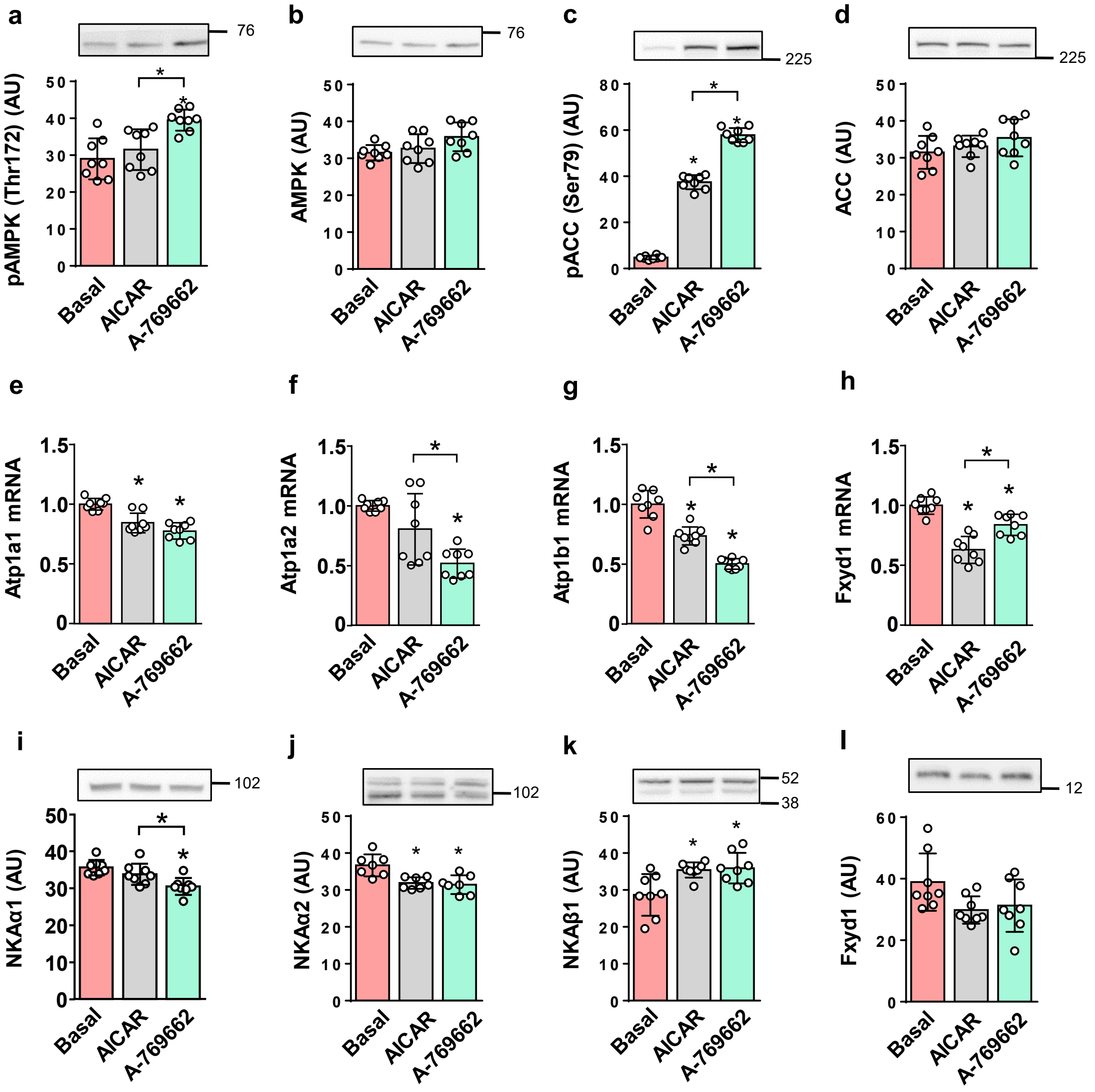
a
b
c
d
76
76
225
225
pAMPK (Thr172) (AU)
AMPK (AU)
pACC (Ser79) (AU)
ACC (AU)
Basal
AICAR
A-769662
e
f
g
h
Atp1a1 mRNA
Atp1a2 mRNA
Atp1b1 mRNA
Fxyd1 mRNA
i
j
k
l
102
102
52
38
12
NKAα1 (AU)
NKAα2 (AU)
NKAβ1 (AU)
Fxyd1 (AU)

Figure 3

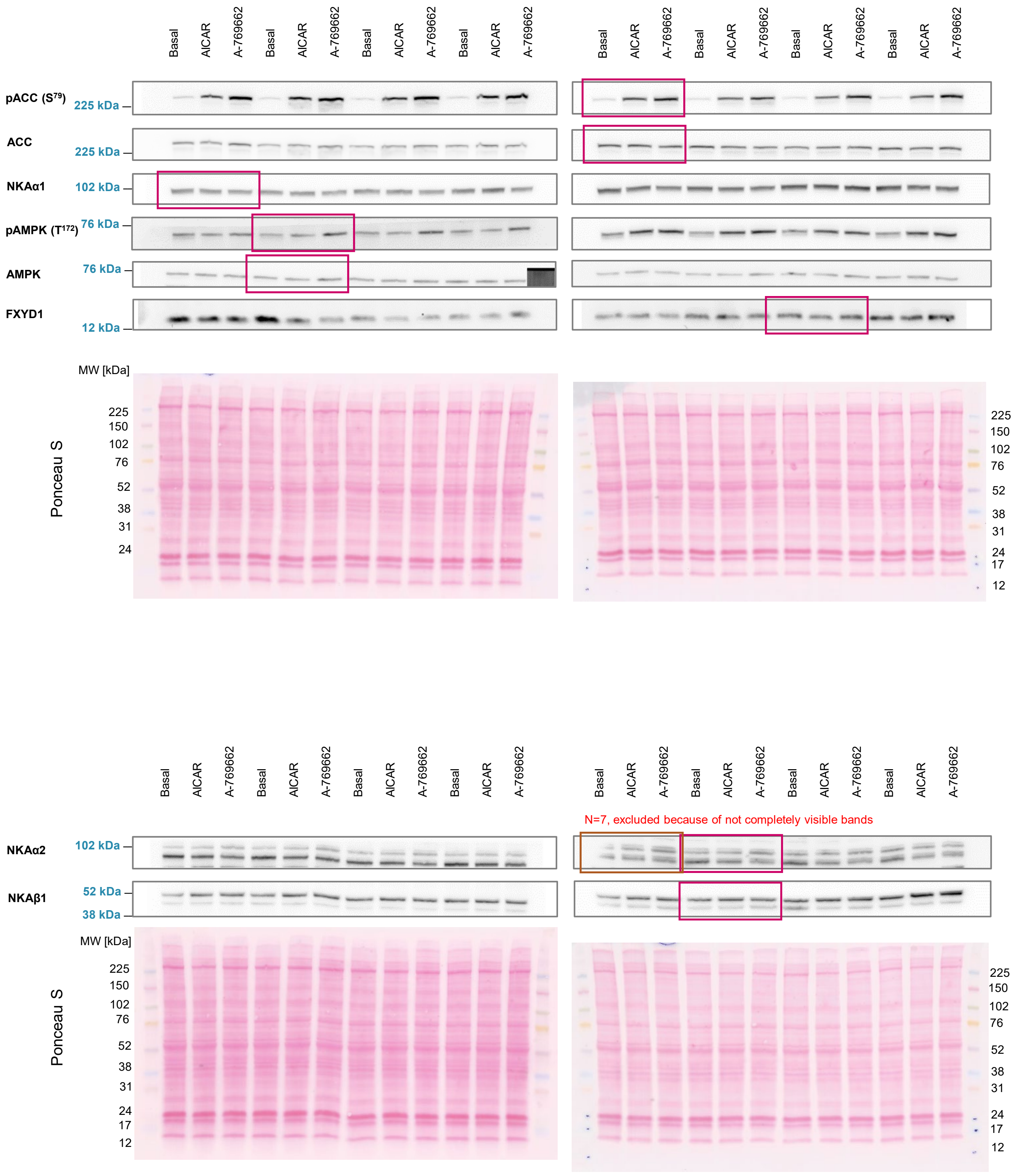

The frame shows the blots that are presented in the figure.

Figure 4

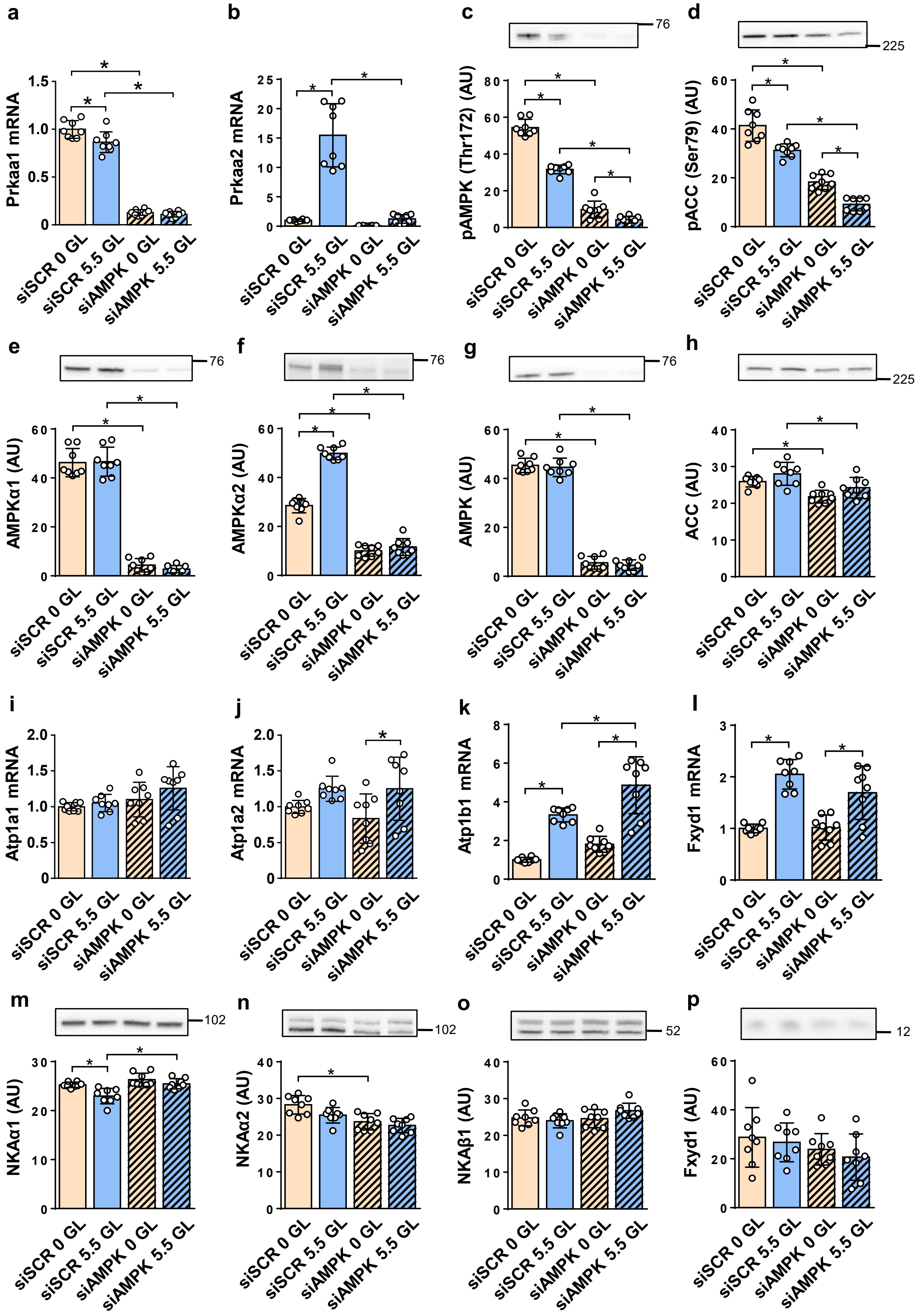

a
Prkaa1 mRNA
b
Prkaa2 mRNA
c
pAMPK (Thr172) (AU)
76
d
pACC (Ser79) (AU)
225
e
AMPKα1 (AU)
76
f
AMPKα2 (AU)
76
g
AMPK (AU)
76
h
ACC (AU)
225
i
Atp1a1 mRNA
j
Atp1a2 mRNA
k
Atp1b1 mRNA
l
Fxyd1 mRNA
m
NKAα1 (AU)
102
n
NKAα2 (AU)
102
o
NKAβ1 (AU)
52
p
Fxyd1 (AU)
12
siSCR 0 GL
siSCR 5.5 GL
siAMPK 0 GL
siAMPK 5.5 GL

Figure 4

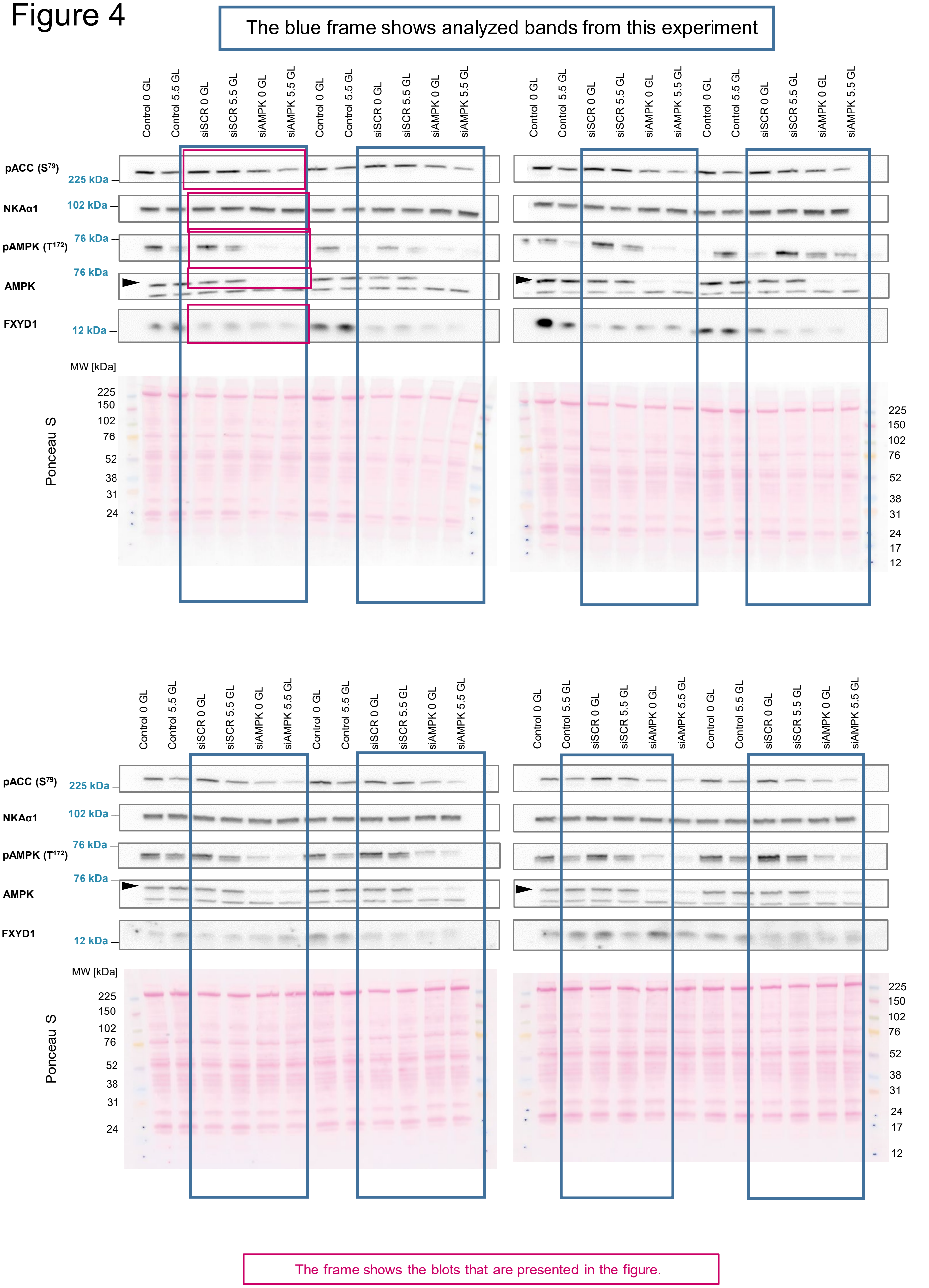
The blue frame shows analyzed bands from this experiment
Control 0 GL
Control 5.5 GL
siSCR 0 GL
siSCR 5.5 GL
siAMPK 0 GL
siAMPK 5.5 GL
pACC (S79)
225 kDa
NKAα1
102 kDa
pAMPK (T172)
76 kDa
AMPK
76 kDa
FXYD1
12 kDa
MW [kDa]
Ponceau S
225
150
102
76
52
38
31
24
17
12
The frame shows the blots that are presented in the figure.

Figure 4

The blue frame shows analyzed bands from this experiment

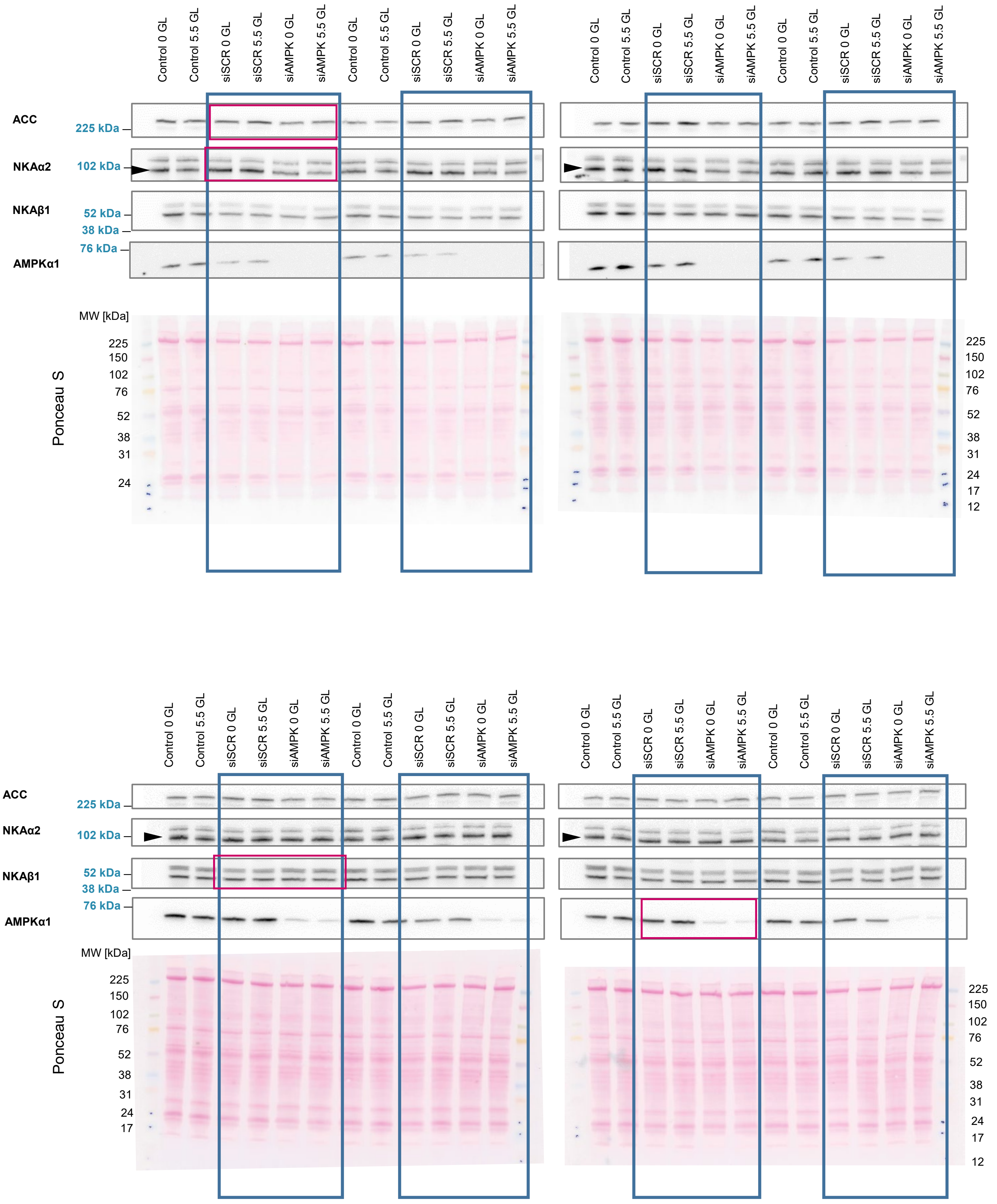

The frame shows the blots that are presented in the figure.

Figure 4

The blue frame shows analyzed bands from this experiment

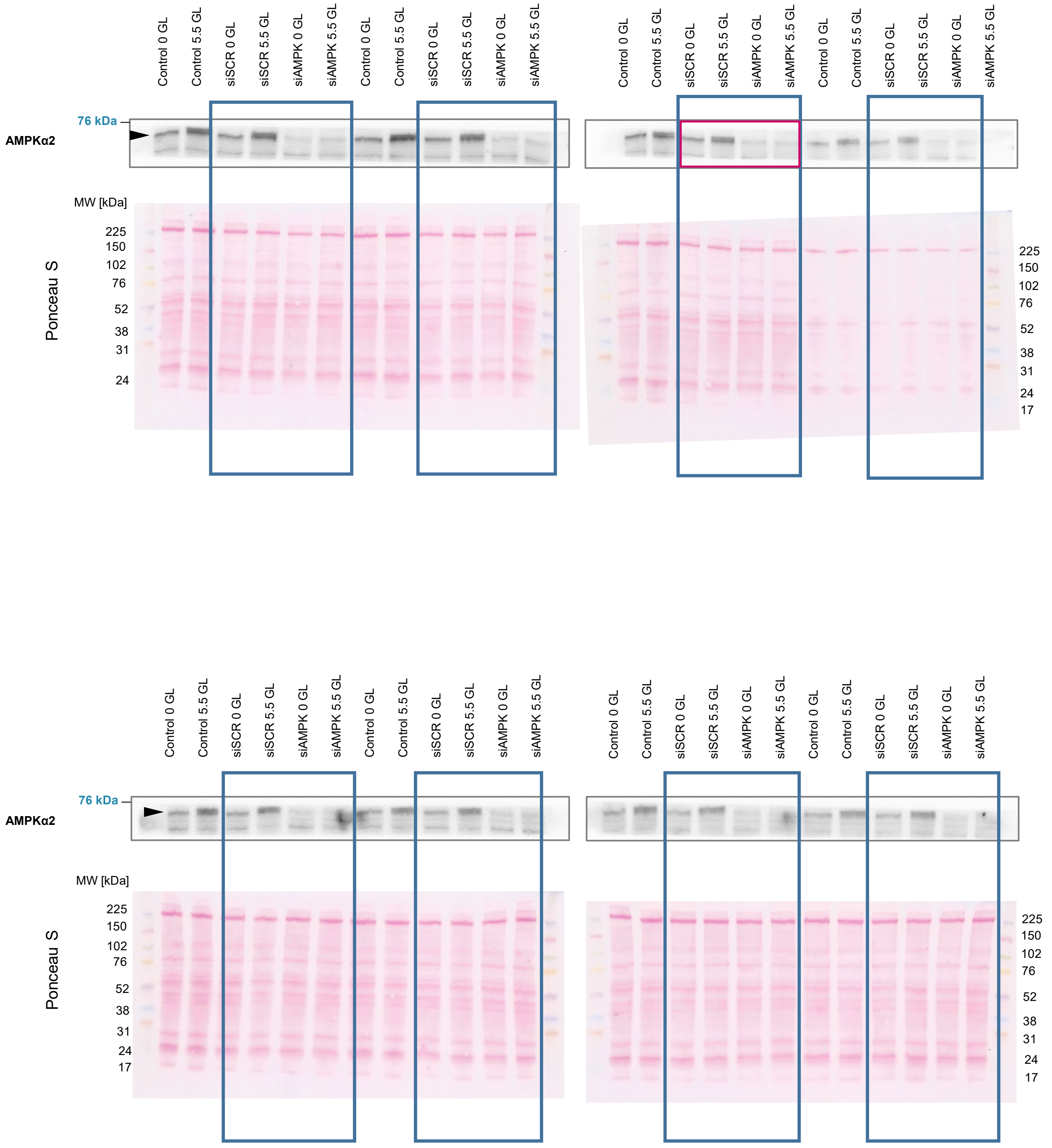

The frame shows the blots that are presented in the figure.

Figure 5

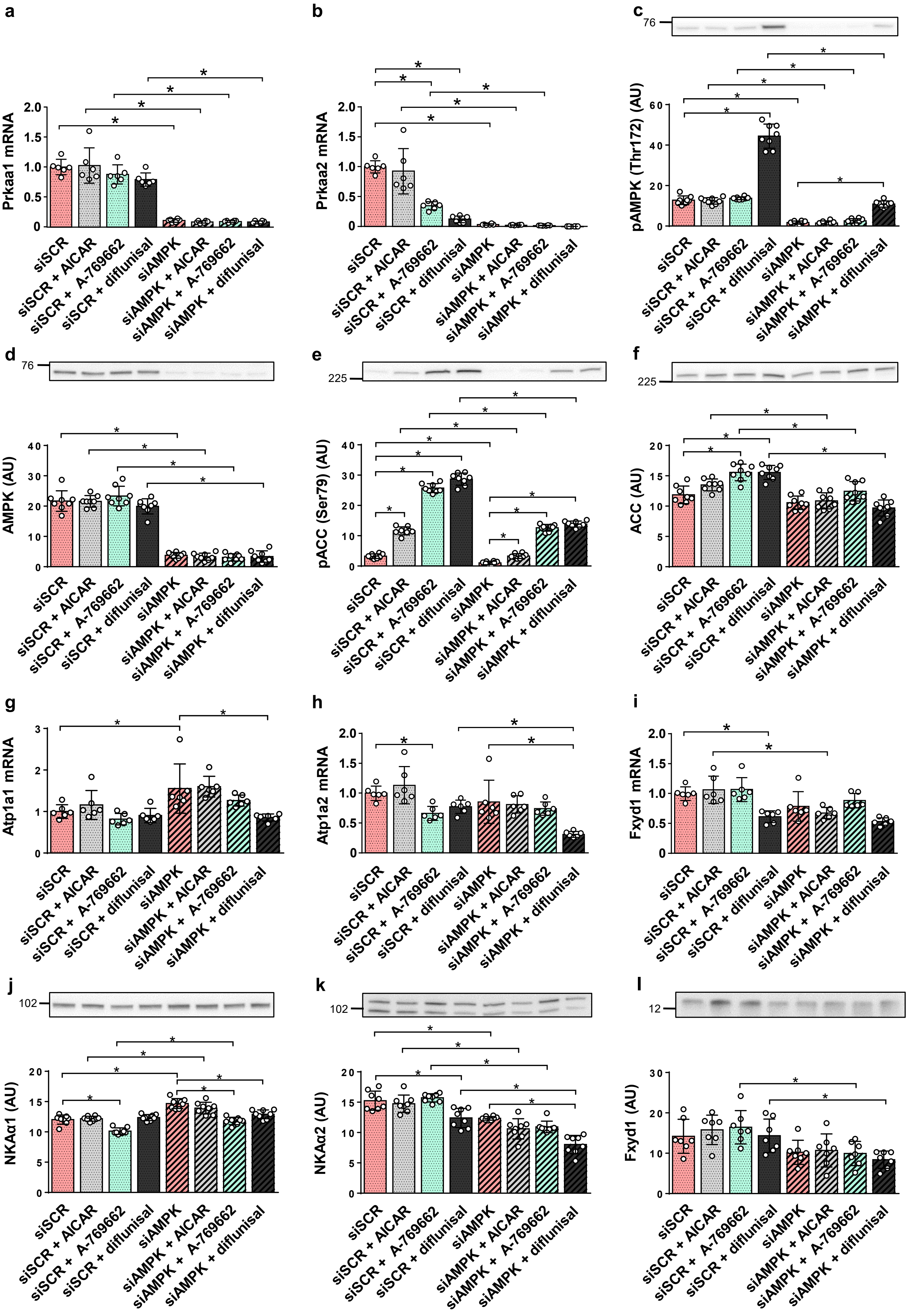

a
Prkaa1 mRNA
b
Prkaa2 mRNA
c
pAMPK (Thr172) (AU)
d
AMPK (AU)
e
pACC (Ser79) (AU)
f
ACC (AU)
g
Atp1a1 mRNA
h
Atp1a2 mRNA
i
Fxyd1 mRNA
j
NKAα1 (AU)
k
NKAα2 (AU)
l
Fxyd1 (AU)
siSCR
siSCR + AICAR
siSCR + A-769662
siSCR + diflunisal
siAMPK
siAMPK + AICAR
siAMPK + A-769662
siAMPK + diflunisal
76
225
102
12

Figure 5

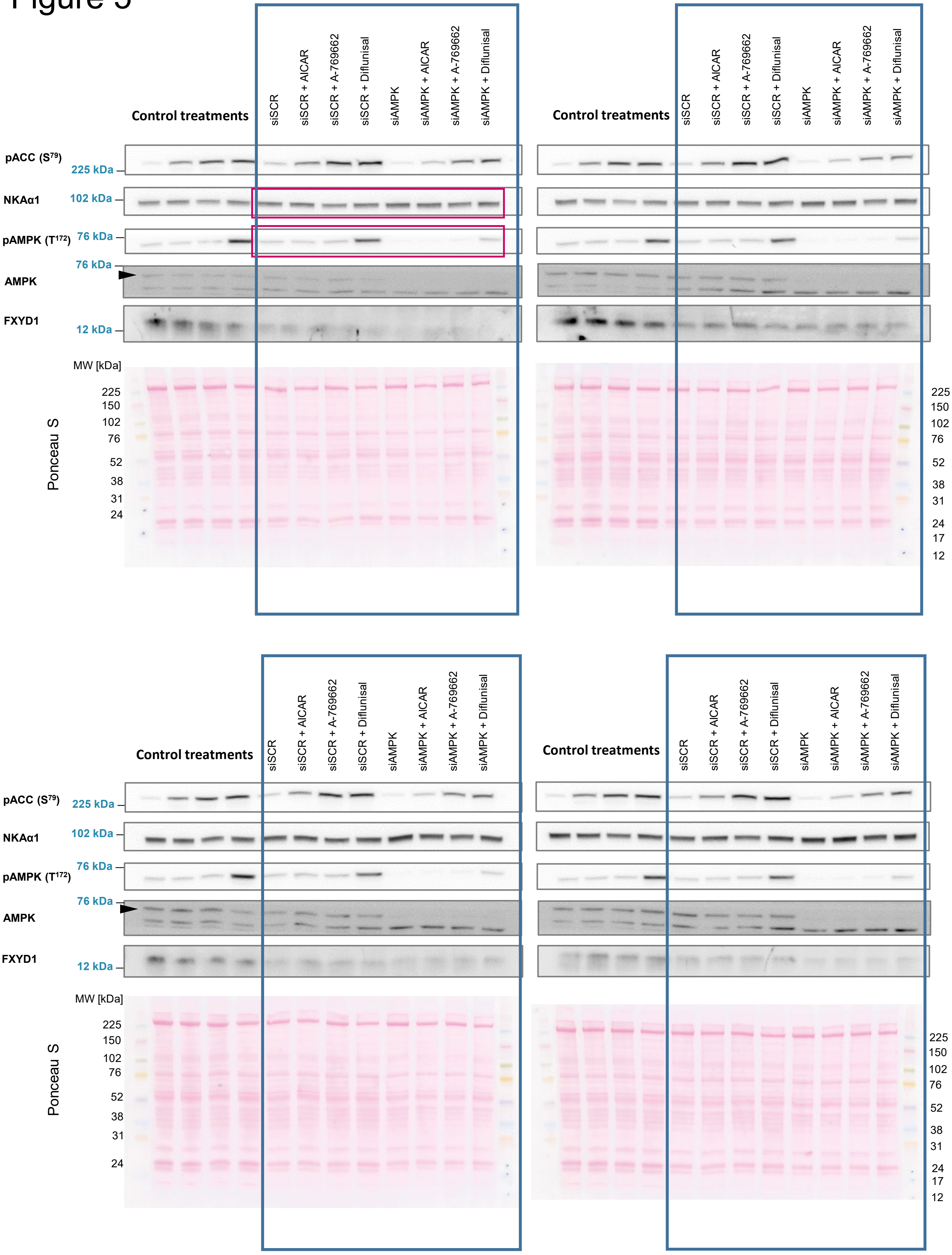

The blue frame shows analyzed bands from this experiment

The frame shows the blots that are presented in the figure.

Figure 5

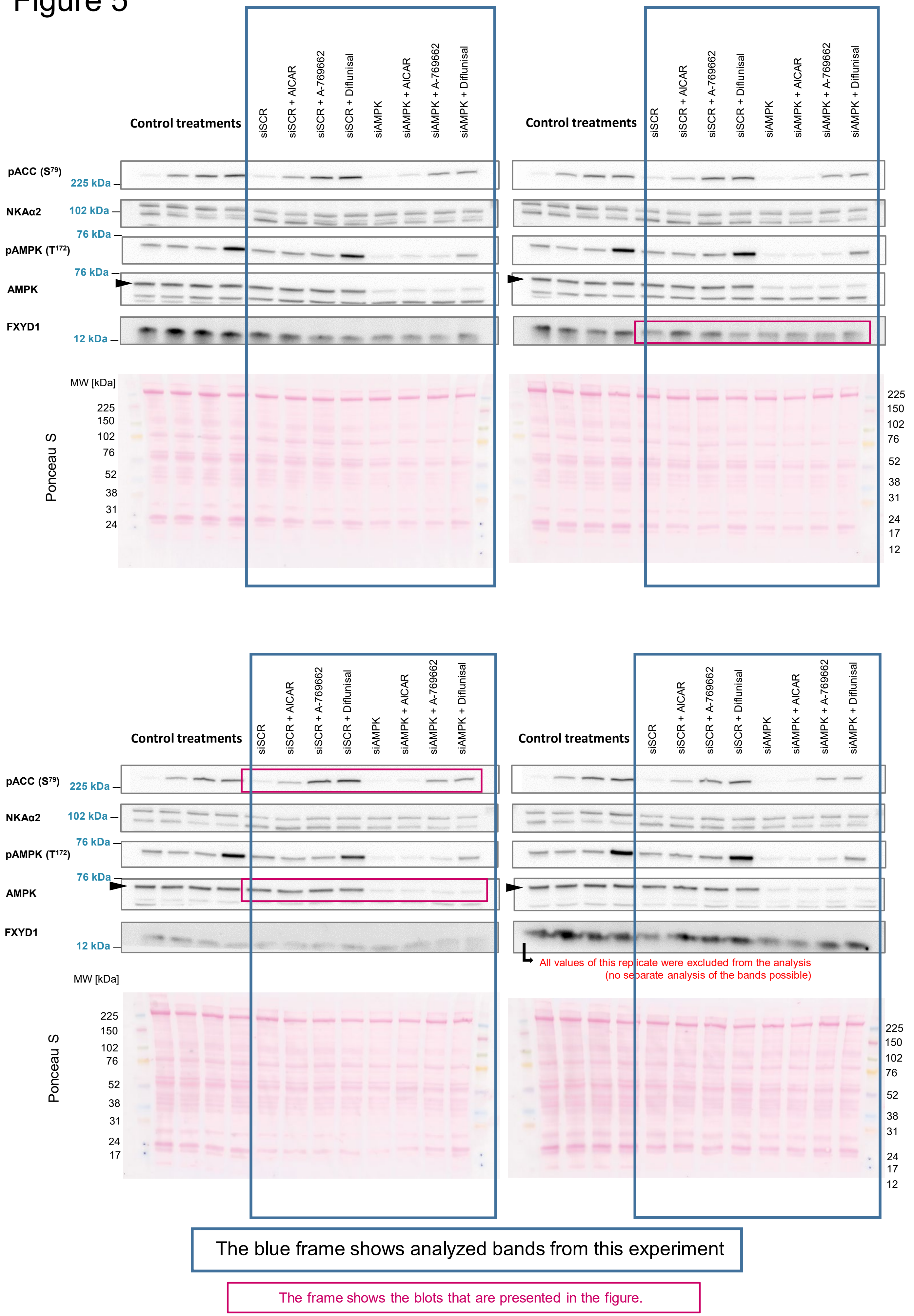
Control treatments
siSCR
siSCR + AICAR
siSCR + A-769662
siSCR + Diflunisal
siAMPK
siAMPK + AICAR
siAMPK + A-769662
siAMPK + Diflunisal
pACC (S79)
225 kDa
NKAα2
102 kDa
76 kDa
pAMPK (T172)
76 kDa
AMPK
FXYD1
12 kDa
MW [kDa]
Ponceau S
225
150
102
76
52
38
31
24
17
12
All values of this replicate were excluded from the analysis
(no separate analysis of the bands possible)
The blue frame shows analyzed bands from this experiment
The frame shows the blots that are presented in the figure.

Figure 5

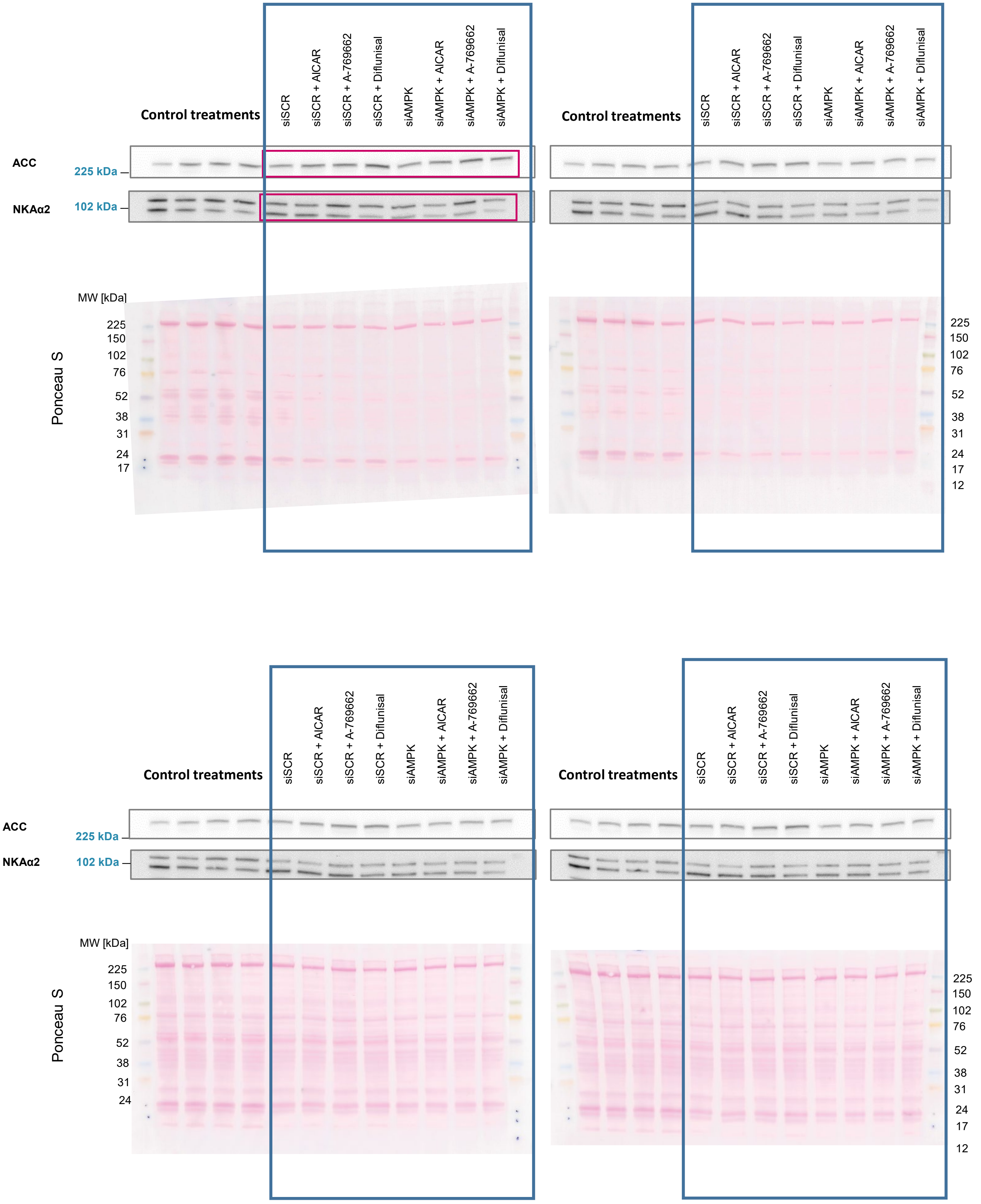

The blue frame shows analyzed bands from this experiment

The frame shows the blots that are presented in the figure.

Figure 5

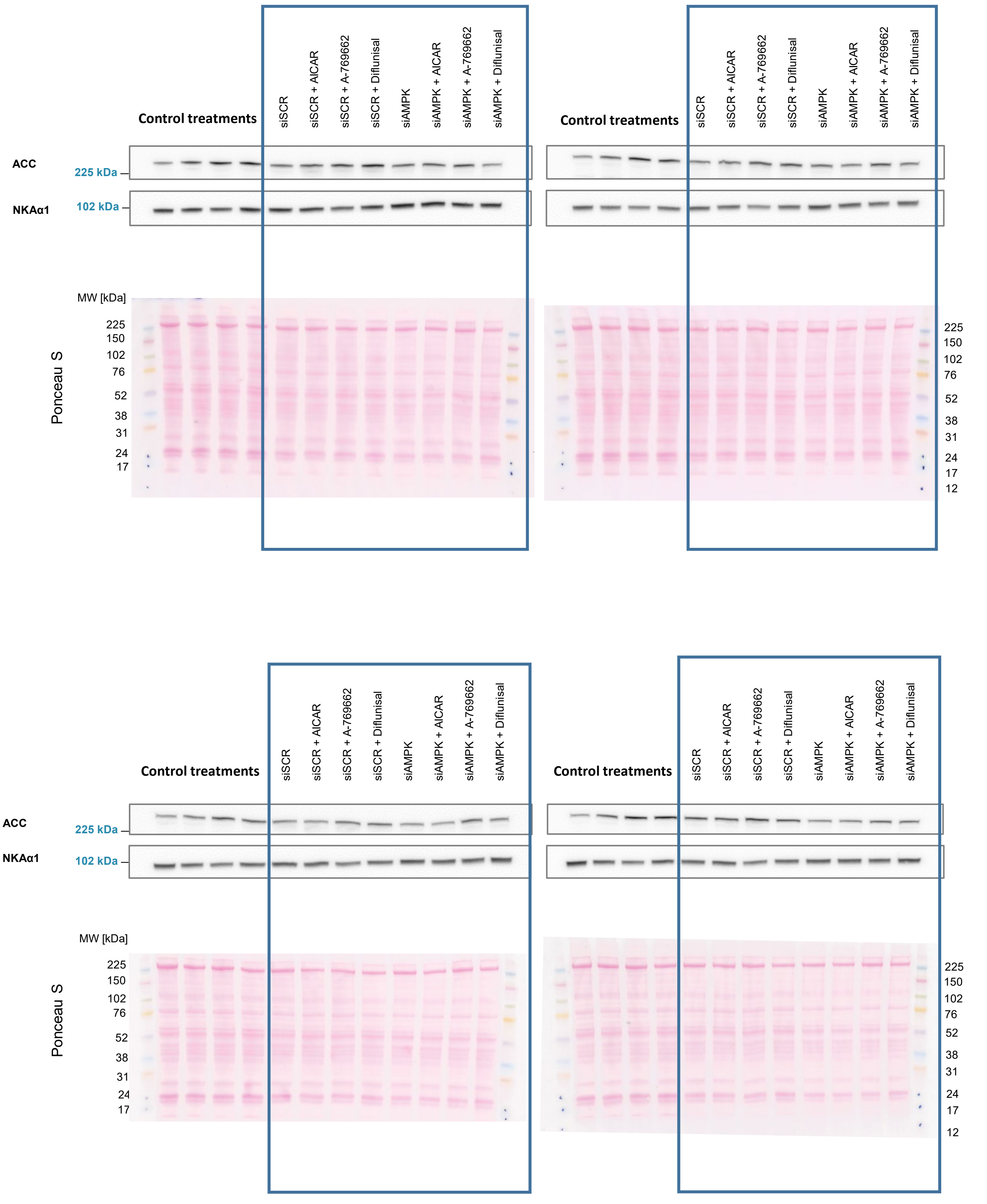

The blue frame shows analyzed bands from this experiment

Figure 6

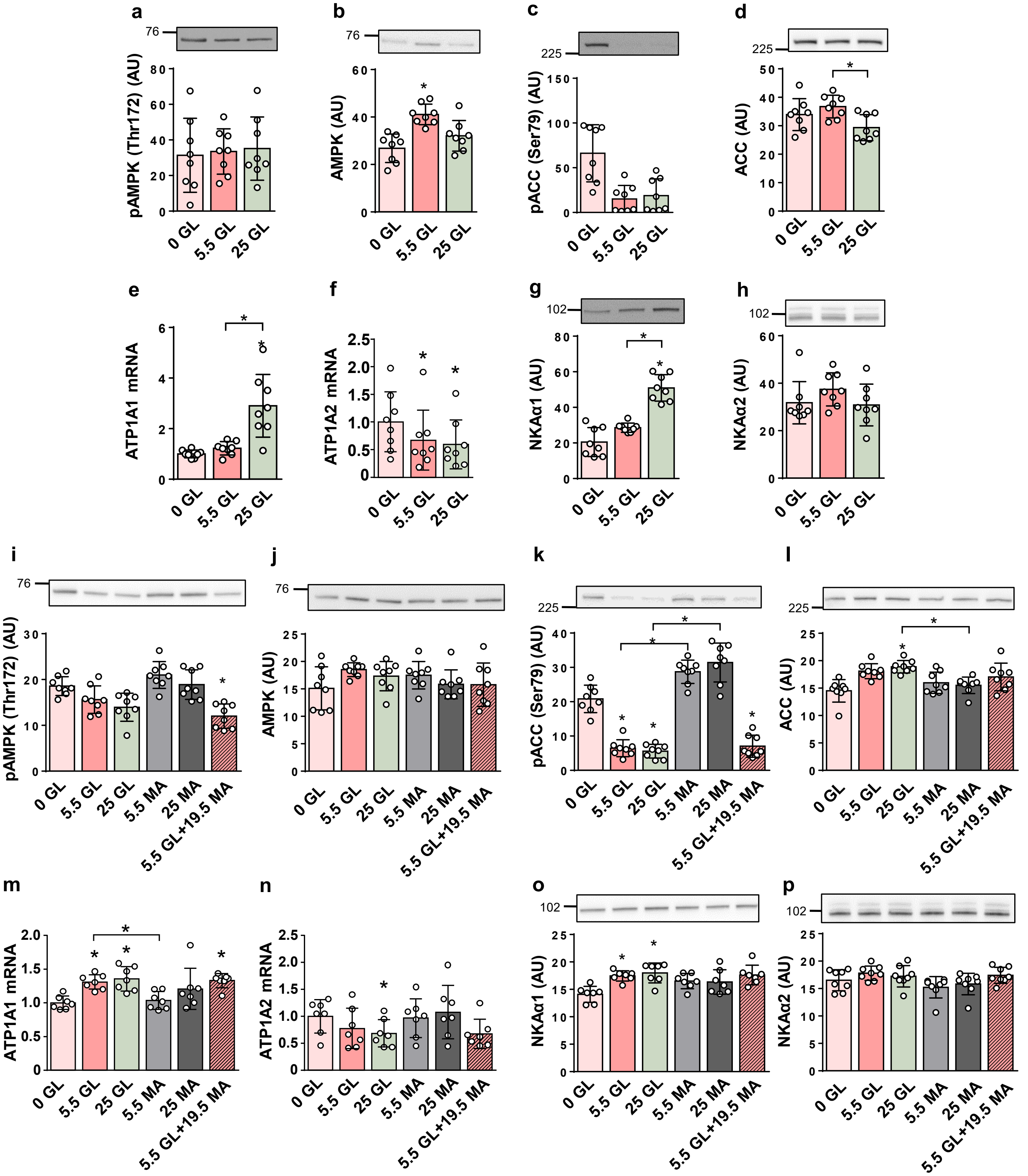

a
b
c
d
e
f
g
h
i
j
k
l
m
n
o
p
pAMPK (Thr172) (AU)
AMPK (AU)
pACC (Ser79) (AU)
ACC (AU)
ATP1A1 mRNA
ATP1A2 mRNA
NKAα1 (AU)
NKAα2 (AU)
0 GL
5.5 GL
25 GL
5.5 MA
25 MA
5.5 GL+19.5 MA
76
225
102

Figure 1a-g

The blue frame shows analyzed bands from this experiment

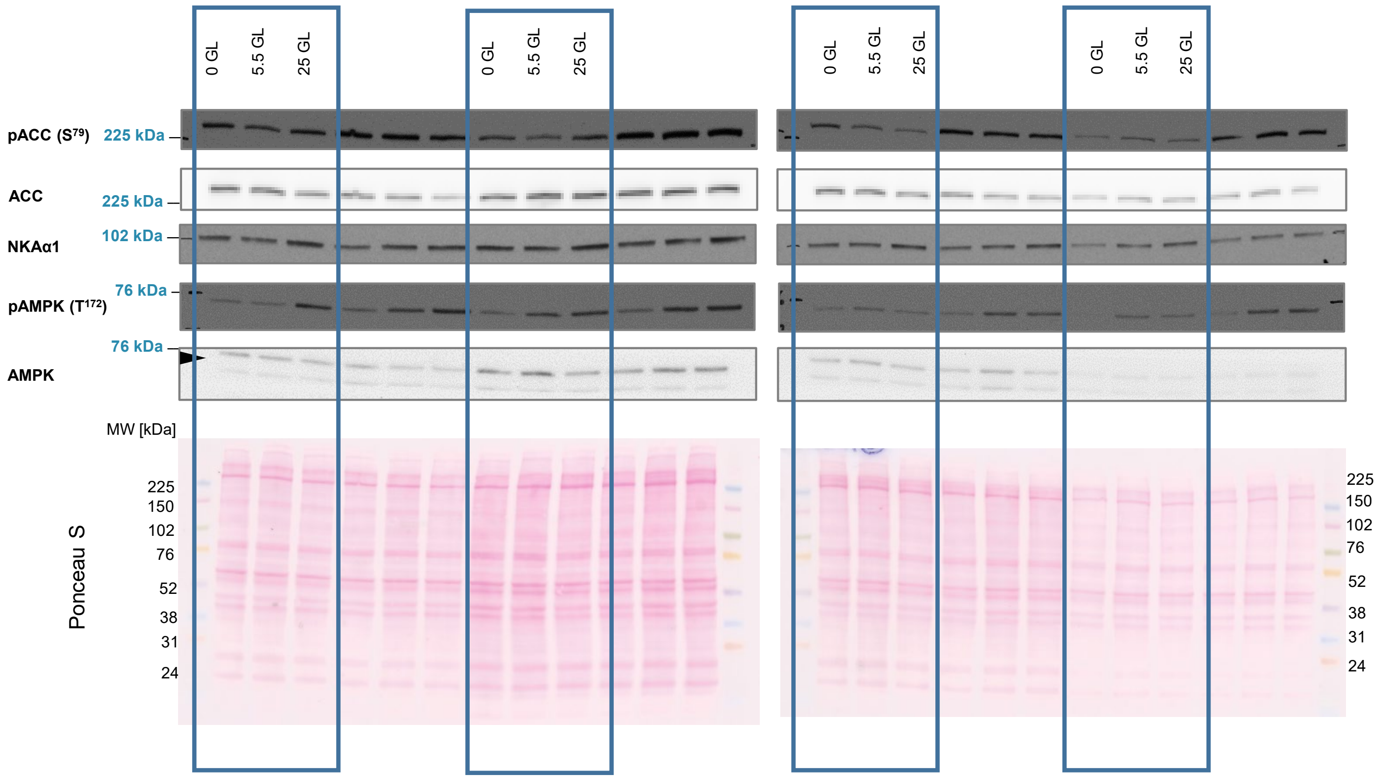

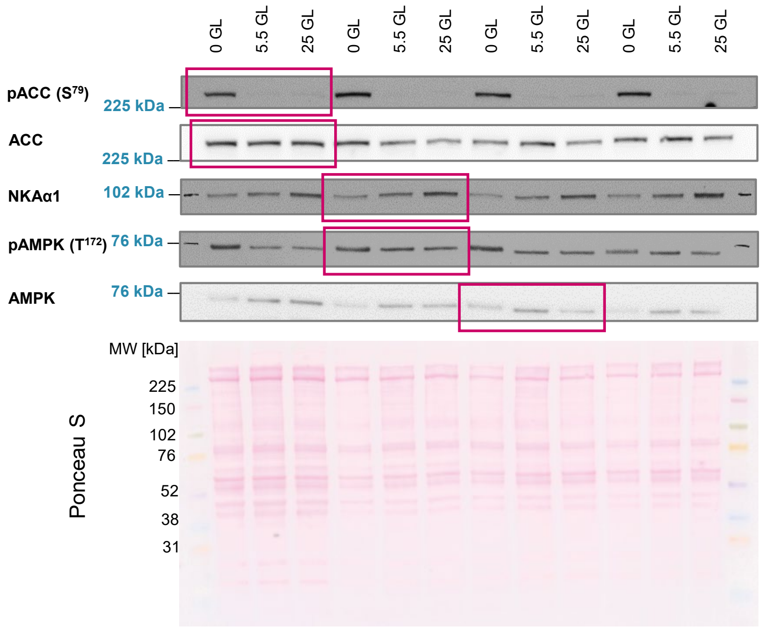

The frame shows the blots that are presented in the figure.

Figure 1h

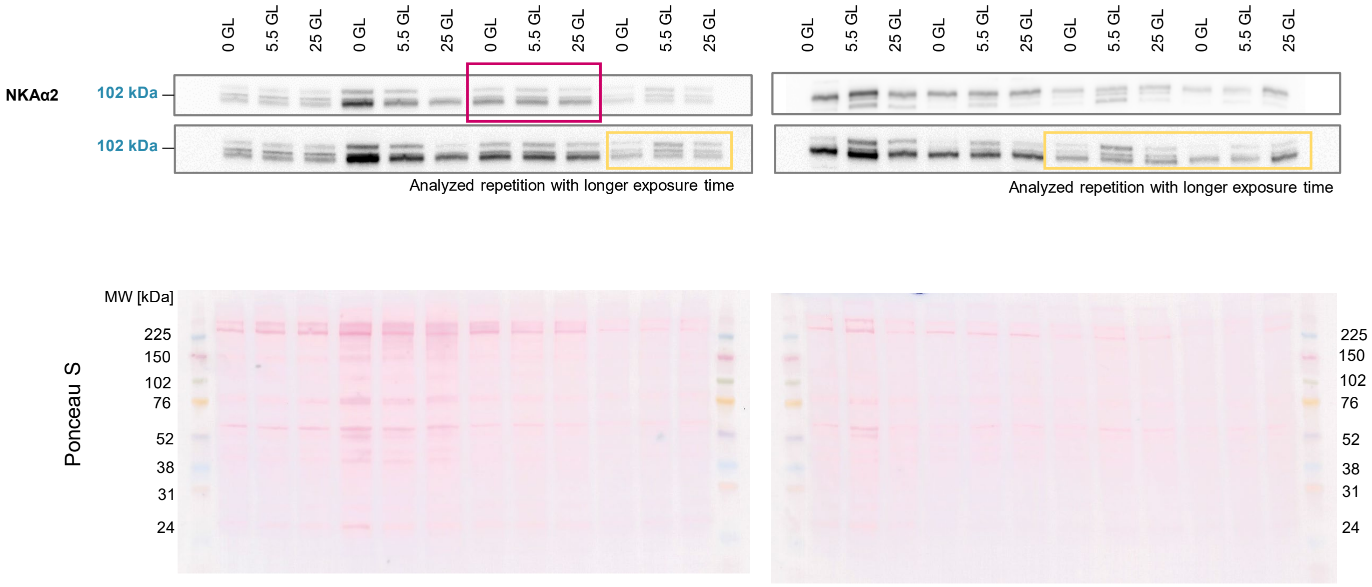

The frame shows the blots that are presented in the figure.

Figure 6i-p

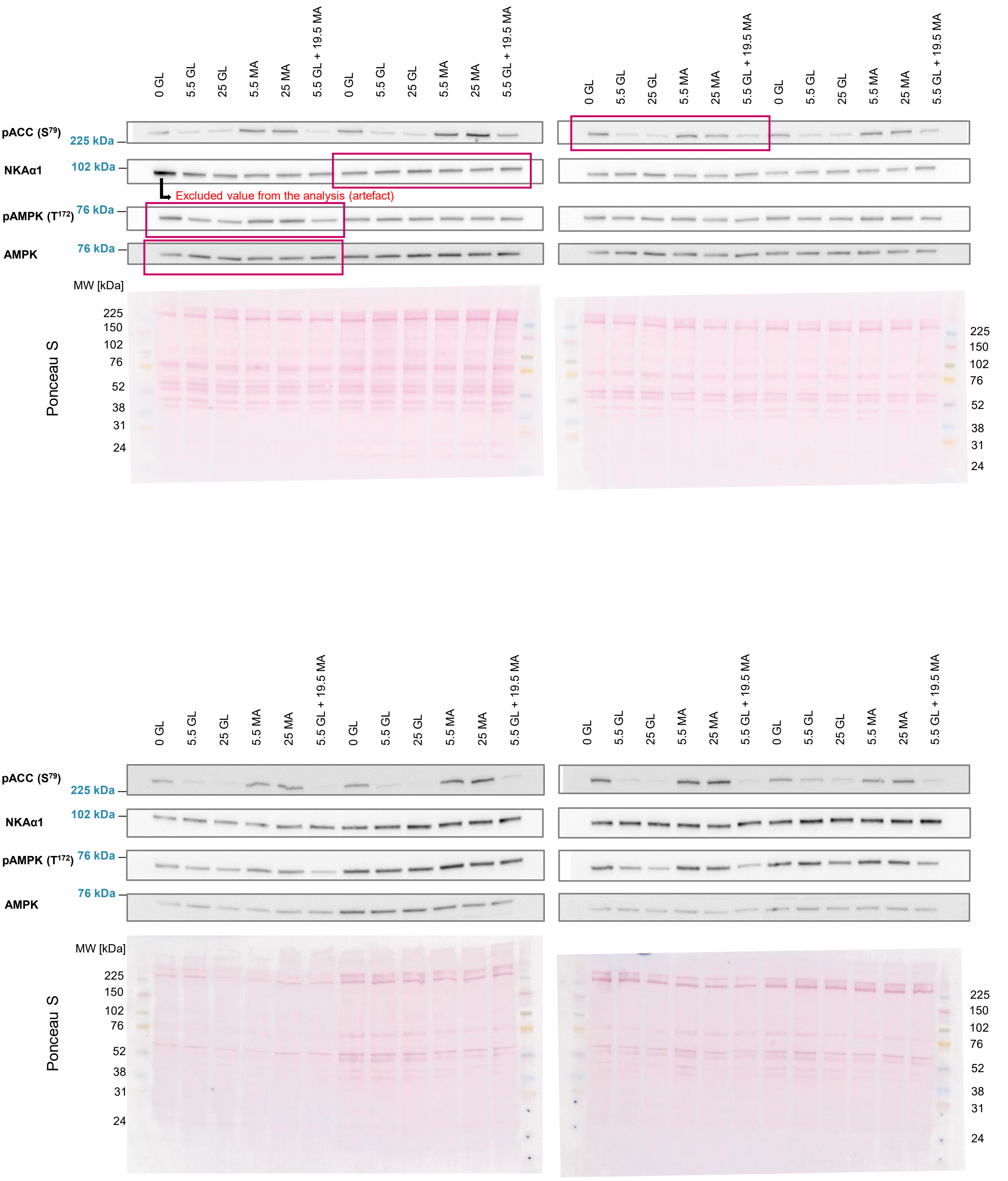

The frame shows the blots that are presented in the figure.

Figure 6i-p

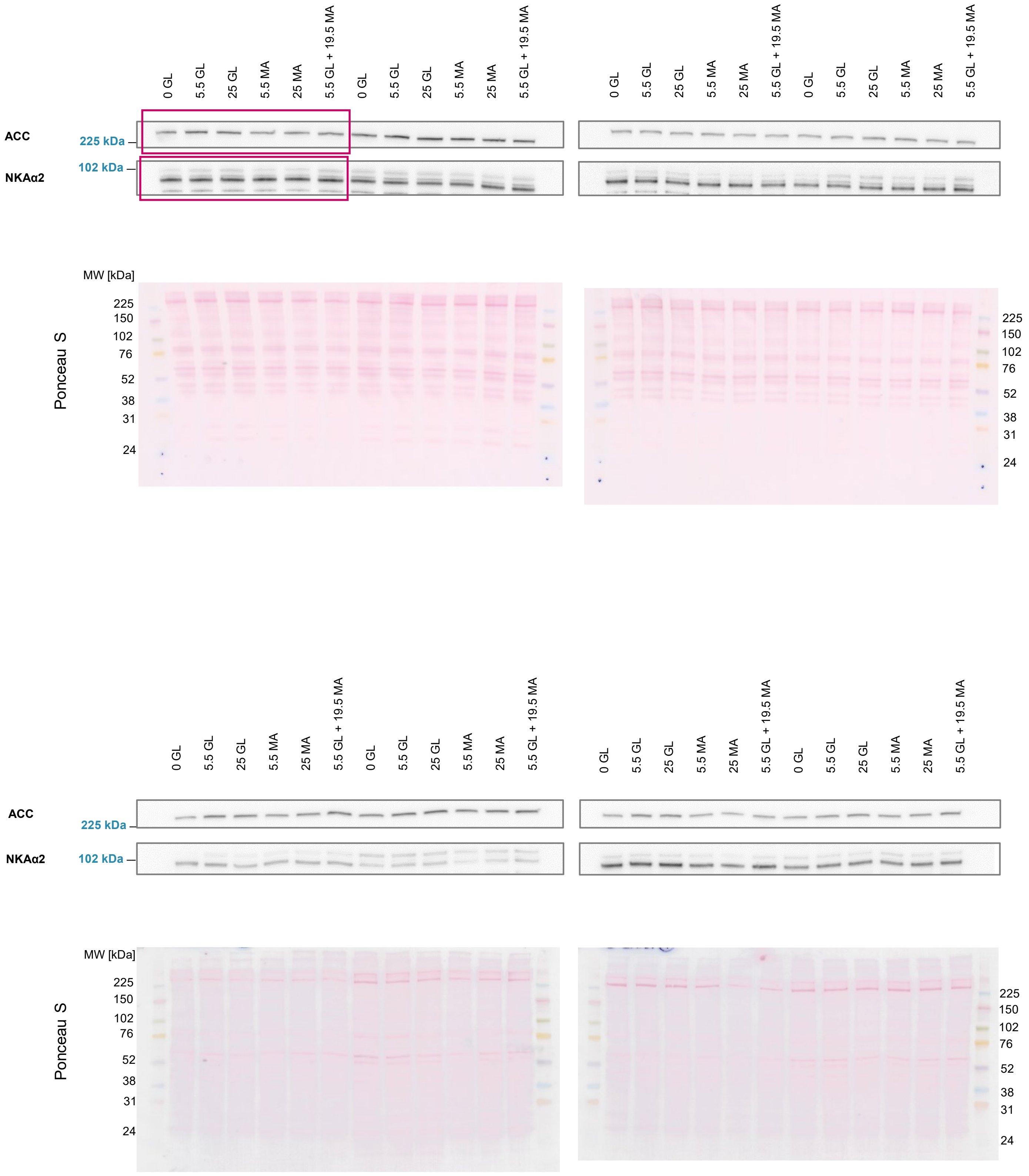

The frame shows the blots that are presented in the figure.

Figure 7

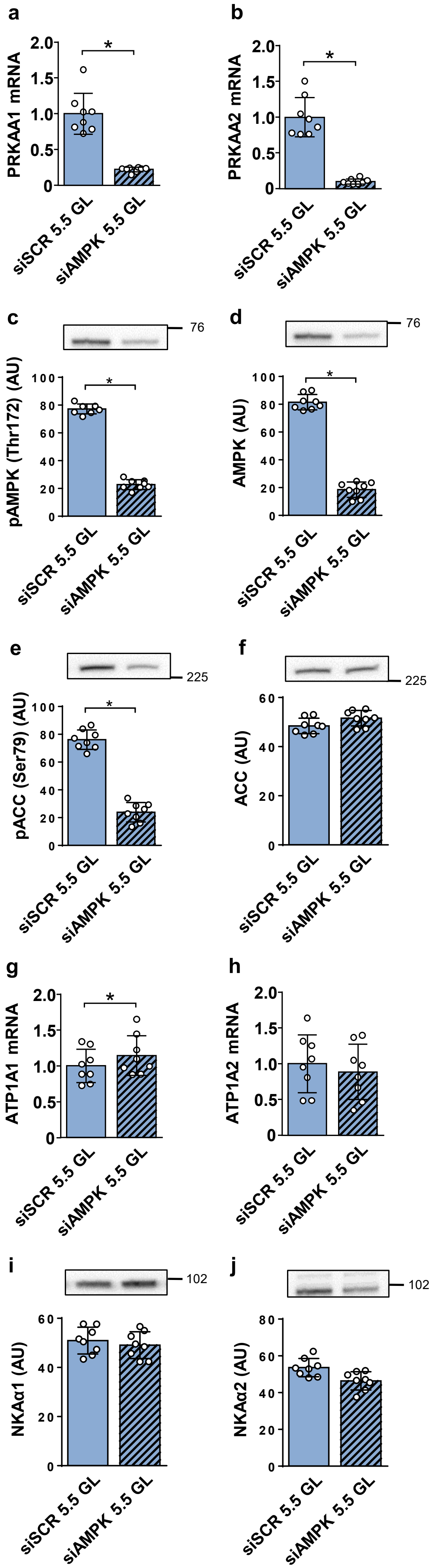

a
PRKAA1 mRNA
*
siSCR 5.5 GL
siAMPK 5.5 GL
b
PRKAA2 mRNA
*
c
76
pAMPK (Thr172) (AU)
d
AMPK (AU)
e
225
pACC (Ser79) (AU)
f
ACC (AU)
g
ATP1A1 mRNA
h
ATP1A2 mRNA
i
102
NKAα1 (AU)
j
NKAα2 (AU)

Figure 7

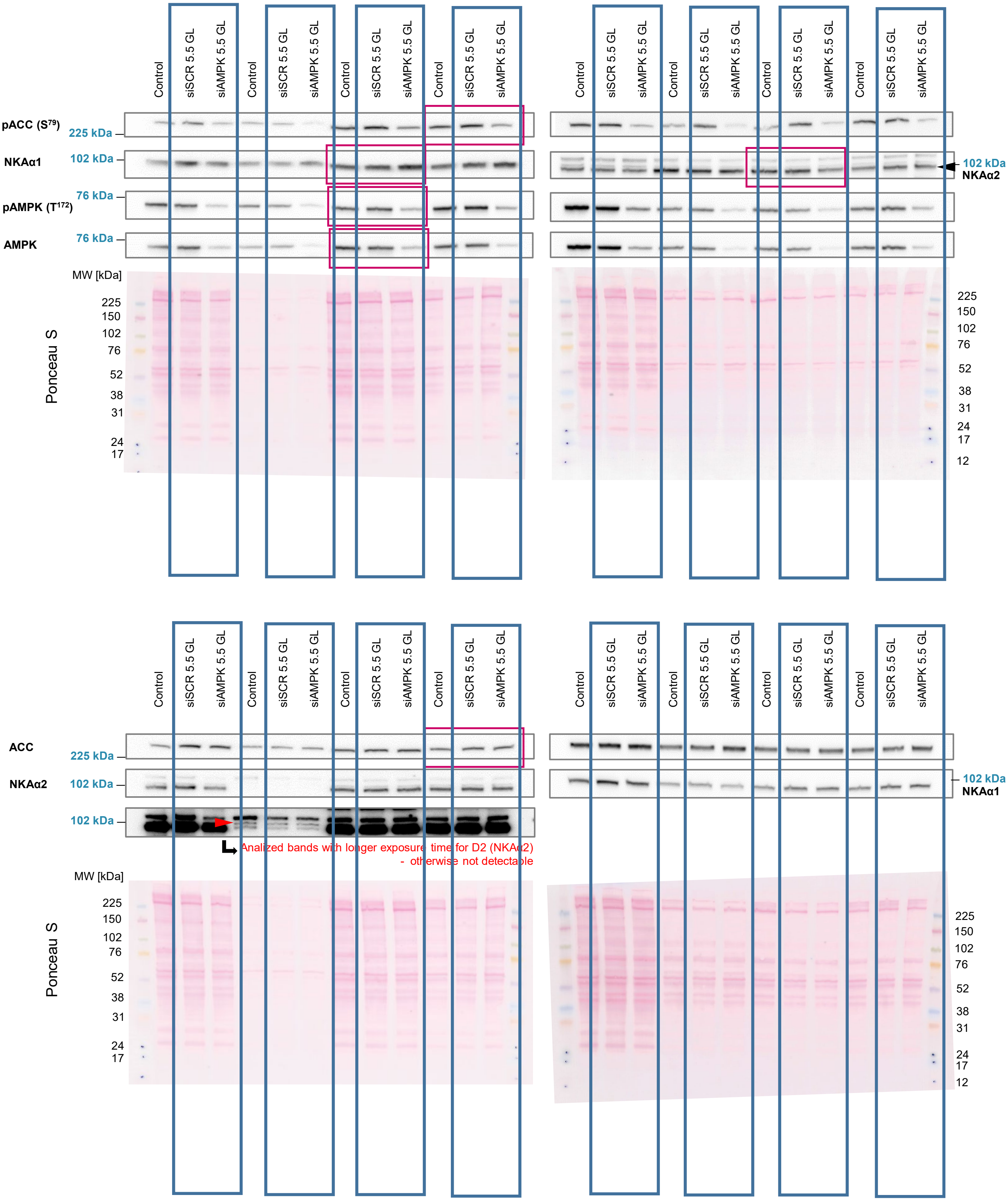

The blue frame shows analyzed bands from this experiment

The frame shows the blots that are presented in the figure.
